# Deep learning identifies TP-41 for methylglyoxal scavenging in Alzheimer's treatment

**DOI:** 10.7150/thno.111550

**Published:** 2026-01-01

**Authors:** Aron Park, Seong-Min Hong, Yeeun Lee, Jungeun Lee, Seunggyu Jeon, Seung-Yong Seo, Jinhyuk Lee, Seon-Hyeok Kim, Eun Ji Ko, Hae Ran Lee, Sang Heon Jung, Munhyung Bae, Min Cheol Kang, Myoung Gyu Park, Seungyoon Nam, Sun Yeou Kim

**Affiliations:** 1 Department of Health Sciences and Technology, Gachon Advanced Institute for Health Sciences and Technology (GAIHST), Gachon University, Incheon, 21999, Korea.; 2 Division of Food Science and Technology, Gyeongsang National University, Jinju, Gyeongnam 52828, Korea.; 3 Department of Genome Medicine and Science, Gachon Institute of Genome Medicine and Science, Gachon University Gil Medical Center, Gachon University College of Medicine, Incheon, 21565, Korea.; 4 College of Pharmacy and Gachon Institute of Pharmaceutical Science, Gachon University, 191, Hambakmoe-ro, Yeonsu-gu, Incheon 21936, Korea; 5 Genome Editing Research Center, Korea Research Institute of Bioscience and Biotechnology (KRIBB), Daejeon 34141, Korea.; 6 MetaCen Therapeutics Company, Changnyong-daero 256 Beon-gil, Yeongtong-gu, Suwon-si, Gyeonggi-do 16229, Korea.; 7 Department of Translational-Clinical Medicine, Gachon Advanced Institute for Health Sciences and Technology (GAIHST), Gachon University, Incheon, 21999, Korea.

**Keywords:** methylglyoxal, Alzheimer's disease, deep learning, memory impairment, drug discovery

## Abstract

**Rationale**: Increased levels of advanced glycation end products (AGEs) have been observed in the brain tissues of patients with Alzheimer's disease (AD). Methylglyoxal (MGO) is a potent precursor of AGEs. To date, there have been no reports of utilizing deep learning (DL) technologies to target MGO scavengers for the development of AD therapeutics. Therefore, DL-driven approaches may play a crucial role in identifying potential MGO scavengers and candidates for Alzheimer's treatment.

**Methods**: We developed "DeepMGO," a novel DL-based MGO scavenging activity prediction model, trained on 2,262 MGO scavenging activity assays from 660 compounds. Using this approach, we identified and validated TP-41 as a potential MGO scavenger in a mouse model of memory impairment.

**Results**: DeepMGO demonstrated robust predictive performance and identified novel compounds with high MGO scavenging activity. TP-41 ameliorated depression symptoms and memory deficits in mouse models.

**Conclusions**: Using DeepMGO, we identified TP-41 as a potential therapeutic agent for AD.

## Introduction

Alzheimer's disease (AD) is a neurodegenerative disorder that poses significant global health challenges, characterized by progressive cognitive decline and memory impairment impacting millions worldwide 1. Despite extensive research efforts into AD pathogenesis, effective treatments remain elusive and current therapies only provide limited symptomatic relief and fail to halt disease progression. AD is becoming increasingly prevalent owing to the aging global population, highlighting the need for more effective interventions. Advancements in research, particularly in targeting the underlying mechanisms such as advanced glycation end products (AGEs), amyloid plaques, tau phosphorylation, and neuroinflammation, are crucial for developing treatments that can slow or prevent the onset of AD 2.

Recent studies emphasize the role of methylglyoxal (MGO), a dicarbonyl compound, in AD pathogenesis 3. MGO induces cellular damage, inflammation, cytotoxicity, and apoptosis through reactive oxygen species (ROS) generation 4, 5. MGO is implicated in cellular damage associated with hallmark features of AD, such as neurofibrillary tangles, amyloid β (Aβ) plaques, and the formation of AGEs 6, 7. Moreover, AGEs are formed during the glycolysis process through the non-enzymatic reaction of dicarbonyl compounds (such as MGO) with amino acids in proteins 8. In individuals with AD, abnormal accumulation of MGO has been observed in various tissues, including cerebral fluids and organs 9-17, suggesting its potential as a therapeutic target.

Despite growing recognition of MGO's role in AD, current therapeutic approaches remain limited. MGO-scavenging strategies have emerged as potential interventions for AD treatment 18, 19, and aim to reduce oxidative stress and inhibit AGE formation associated with MGO. Compounds such as aminoguanidine, tryptophan (Trp), tryptamine, and 5-hydroxytryptophan (5-HTP) have shown promise in this regard 20, 21.

Deep learning (DL) algorithms have been applied to screen vast libraries of compounds to identify new drug candidates targeting AD 22. DL has been successfully used in the identification of small molecules capable of modulating the acetylcholinesterase enzyme, which plays a key role in AD pathology, leading to the discovery of several promising drug leads 23. Therefore, we aimed to identify the most efficient MGO scavenger molecules using a novel DL technique.

The majority of DL models utilize large assay data from chemical libraries to facilitate virtual drug screening and prioritization of candidate compounds 24. For MGO scavenger identification, constructing a DL model using a dataset derived from chemical properties and MGO scavenging activity assays holds great potential for the rapid identification of promising novel MGO scavengers while minimizing the need for extensive assays on numerous new compounds.

To address this knowledge gap, our study introduces an innovative approach using DL to develop an MGO scavenging activity screening prediction model called "DeepMGO." In addition, we evaluated and validated the predictive performance of our model using an independent test set collected from the assay results of diverse publications. We used this novel DL model to prioritize previously unknown compounds as application data for identifying TP-41 as a novel MGO scavenger derived from Trp. We further sought to validate findings from the DL model and investigated the top candidate molecules' impacts on AD-related phenotypes in mouse models exposed to high-dose MGO 2.

## Methods

### MGO scavenging activity assay

MGO scavenging activity assay was performed to analyze the interactions between MGO and compounds during 0, 1, and 6 days, according to the protocol of Nemet *et al*. with slight modification 25. MGO in the presence (or absence) of 660 compounds (natural compounds [482 species], FDA-approved drugs [159 species], and amino acids [19 species]) were incubated in PBS at pH 7.4 and 0.02% sodium azide for 1 week at 37 ^°^C. The affinity of the combined MGO and compound was evaluated by measuring the fluorescence intensity at excitation/emission wavelengths of 355/460 nm using a VICTOR^TM^ X3 multilabel plate reader (PerkinElmer, MA, USA).

### In-house data for construction of MGO scavenging activity prediction model

The in-house data consisted of 2,262 MGO scavenging activity values from MGO scavenging activity assays, including 660 compounds at concentrations ranging from 0.001 to 1,000 μM (Figure S1). Molecular feature calculations were performed for these data using the PaDEL Descriptor 26. Specifically, molecular feature vectors of compounds were determined by utilizing the simplified molecular-input line-entry system format of the compounds as input. The min-max normalization method was employed to process individual molecular features, while Z-normalization was used to standardize the MGO scavenging activity values. The Z-normalized MGO scavenging activity value was considered the MGO scavenging activity score. The in-house data were divided into training, validation, and test datasets at a ratio of 8:1:1 (Figure S1). The higher score indicates a stronger affinity.

### Construction of MGO scavenging activity prediction models using DL

A convolutional neural network architecture was employed to predict the MGO scavenging activity and generate a DL algorithm, named DeepMGO. Convolutional layers and deep neural network (dense) layers were used for molecular features, with a concatenated dense layer for screening the concentration of the last layer of molecular features. From the concatenation of the molecular features and screening concentrations, we added two dense layers to achieve the predicted output (Figure S2 and Table S1).

Herein, we assume that for DeepMGO, *x* is the input for each layer of the convolutional network and *conv(x)_jm_* is the output of the layer, where *j* is the index of the output position and *m* is the index of the kernels.


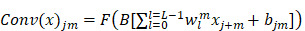

(Eq. 1)

In Eq. 1, *b_jm_* is a bias term, *w^m^_l_* the *m*-th weight in the *l*-th kernel tensor,* L* represents the tensor size, *B*(·) is a batch-normalization function, and *F*(·) is an activation function 27, 28.

The performance of the DeepMGO architecture was compared to that of the DeepIC50 29 and ResNet18 30 models. A concatenation layer was added between the screening concentration layer and the last convolutional layer for the molecular features (Tables S2 and S3 for DeepIC50 and ResNet18, respectively).

The parameter options in DeepMGO, DeepIC50, and ResNet18 were set to 200 for the training epoch and 50 for the batch size. The learning rate was set to 0.0002 using the Adam optimizer. As the prediction was regression-based, the root mean square error (RMSE) was used as a loss function, and the activation functions were either the hyperbolic tangent or rectified linear activation (ReLU) functions. All DL models were generated using the keras v2.1.0 package in a Python environment.

### Construction of MGO scavenging activity prediction models using machine learning (ML)

For the ML models, we utilized lasso, ridge, random forest (RF), and support vector regression (SVR), and each ML method employed the scikit-learn Python package. The optimal hyperparameters for the lasso, ridge, RF, and SVR were selected while searching for the best performance in a set of hyperparameter values (Table S4).

The hypopt Python package was used for hyperparameter optimization with the training and validation datasets. Using DeepMGO, the MGO-scavenging effects of various Trp derivatives were predicted, with further analysis of the compounds' structure-activity relationships.

### Feature selection

In the training dataset, the feature importance was evaluated using univariate linear regression tests. Hence, from all the features, we selected the top 10%, 30%, 50%, 70%, and 90% important features. DL and ML models were built using the training dataset according to the selected features.

### Evaluation of DeepMGO using an independent test dataset

Additional validation of DeepMGO was performed using an independent test dataset comprising 61 compounds from literature sources that were identified as being in 50 active and 11 non-active states to bind MGO or AGEs (Table S5). The molecular features of each compound were determined using the PaDEL Descriptor. The screening concentration for DeepMGO was set at 400 µM, which was the screening concentration in the in-house data.

### Metrics for performance comparisons

To compare the performance of the MGO scavenging activity prediction models, the RMSE and R^2^ were calculated using the predicted and observed MGO scavenging activity scores in the test set as follows:

RMSE =
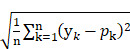

(Eq. 2)

R^2^ = 
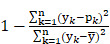

(Eq. 3)

where *n* is the number of cases, *y_k_* is the *k*-th observed MGO scavenging activity score, and *p_k_* is the predicted MGO scavenging activity score for the *k*-th case. Thus, 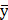
is the overall mean of all *y_k_*'s.

The RMSE was transformed to log_2_(RMSE). The calculated R^2^ and log_2_(RMSE) values were visualized as an integrated heatmap with a dot plot using the ggplot and ggpubr packages in R. Scatter plots and Pearson's correlation coefficient (PCC) values were generated using GraphPad Prism (version 10). For model validation using the independent dataset, we calculated the area under the receiver operating characteristic curve (AUROC) and the optimal cutoff value *via* the multipleROC package in R.

### Identification of TP-41 as a putative MGO scavenger for AD using application data

For the application data consisting of previously unknown compounds, we generated the molecular features of 40 novel and 4 putative MGO scavengers for AD treatment (Figure S3).

### *In silico* prediction of blood-brain barrier (BBB) permeability

To evaluate the potential of TP-41 and the reference compounds (5-HTP and 5-HT) as known to cross the BBB, we performed an in-silico prediction by the BBB permeability prediction model in LogBB_Pred 31 and ADMET-AI 32. The logBB_Pred model gives predicted logBB value (logBB > -1, BBB permeable). The ADMET-AI provides a BBB permeability score, where a score closer to 1 suggests higher permeability.

### An eXplainable AI (XAI) analysis using SHapley Additive exPlanations (SHAP)

To understand which features the model used for the MGO scavenging activity prediction, we employed SHAP for XAI technique 33, 34. We utilized a model-agnostic KernelExplainer from the shap library in Python. The explainer was initialized with a background dataset comprising 100 instances randomly sampled from the training data to serve as a baseline for predictions. Subsequently, we calculated SHAP values for each feature across the entire test set to evaluate the model's behavior on unseen data. The global importance of each feature was quantified by calculating the mean of the absolute SHAP values across all test instances.

### Data acquisition in UHPLC-qTOF-MS analysis

The diluted samples were centrifuged, and the clear supernatant was used for analysis. 2 μL aliquots were injected. HRMS data were obtained using an Agilent Revident LC coupled to an Agilent Revident LC/Q-TOF MS (G6575A; Santa Clara, USA). Mass spectrometry analysis and chemical profiling were carried out in positive electrospray ionization (ESI) with full-scan MS1, using an m/z 20-1700 scan range. UHPLC runs were performed on an Agilent ZORBAX RRHD Eclipse Plus C_18_ column (2.1 × 100 mm, 1.8 μm; PN 959758-902) set at 40 °C. The mobile phases were A = water + 0.1% formic acid and B = acetonitrile + 0.1% formic acid at a flow rate of 0.30 mL min⁻¹ with the following gradient: 0.0-0.5 min, 10% B (A 90%/B 10%) → 10.0 min, 100% B (A 0%/B 100%) → 12.0-15.0 min, 10% B (re-equilibration). The ESI source was operated at a gas temperature of 320 °C with a drying gas flow of 10 L min⁻¹; the nebulizer was set to 35 psig; the sheath gas temperature and flow were 350 °C and 11 L min⁻¹, respectively; and the capillary voltage was 3500 V.

### Data processing in UHPLC-qTOF-MS analysis

Raw LC-QTOF data were processed in Agilent MassHunter Qualitative Analysis (Agilent Technologies). Analyses used MS1-only data. Extracted-ion chromatograms (EICs) were generated with a extraction window under positive ion mode for the following exact masses: TP-41 (*m/z* 378.17), the proposed TP-41-MGO Schiff-base intermediate (*m/z* 486.21), and a rearranged product (*m/z* 484.15). For the positive-control experiment, EICs were additionally generated for tryptophan (*m/z* 205.05) and a putative Trp-MGO adduct (*m/z* 254.01). Comparative assessments were performed across TP41 (or tryptophan) alone, Day0 (immediately after mixing with MGO), and Day3. Retention-time windows and intensity scales used for plotting matched those reported in the corresponding figure legends (Figure S4).

### MGO-induced memory impairment mouse model

Institute of Cancer Research (ICR) mice (7-weeks-old, male) were obtained from Orient Bio Inc. (Gyeonggi-do, Korea) and acclimated for one week before the start of the experiments under 12/12 h light/dark cycles (temperature of 23 ± 1 °C and 60 ± 5% humidity). The mice were fed a laboratory diet (AIN-76A) and provided with water *ad libitum*. After adaptation, the mice were randomly divided into four groups (five mice per group): control (CON), MGO-treated (MGO, 60 mg/kg), MGO-co-treated with TP-41 (40 mg/kg/d, TP-41), and MGO-co-treated with Trp (40 mg/kg/d). MGO (dissolved in 30% v/v glycerol in pH 7.4 PBS, Sigma, St. Louis, MO, USA) was administered to twice per week at 60 mg/kg* via* rectal injection, following the protocol of Md Samsuzzaman et al. (2024), which demonstrated stable systemic absorption of MGO through rectal delivery in mice 2. TP-41 and Trp (Sigma, St. Louis, MO, USA) were dissolved in saline and orally administered daily for 2 weeks. Mice were randomly assigned to experimental groups. All behavioral scoring and histological quantification were performed by investigators blinded to the treatment conditions. All animal experiments were conducted in accordance with the Care and Use of Laboratory Animals guidelines and approved by the IACUC of Gachon University (approval no. GU1-2022-IA0046).

### 5xFAD mouse model

The male 5xFAD transgenic mouse line (10 weeks old at purchase; B6SJL-Tg(APPSwFlLon, PSEN1*M146L*L286V)6799Vas/Mmjax; The Jackson Laboratory, USA) was maintained by crossing hemizygous 5xFAD males with B6SJL F1 females. After a 2-month treatment period with MGO, TP-41, or Trp, animals were analyzed at 18 weeks of age (approximately 4.5 months). Mice were housed under standard conditions (22 ± 2 °C, 50-60% humidity, 12-h light/dark cycle) with free access to food and water. Experimental groups included WT and 5xFAD animals treated with vehicle, MGO, TP-41, or Trp (n = 5-7 per group). Mice were randomly assigned to experimental groups, and all behavioral scoring and histological quantification were performed by investigators blinded to the treatment conditions. Behavioral tests were performed in the following order, with a minimum 24 h interval between tests to avoid carry-over effects: NORT → Y-maze → Barnes maze. All procedures were approved by the IACUC of Gachon University (approval no. GU1-2025-IA0008).

### Open field test (OFT)

For assessing the locomotor, exploratory and anxiety-like behaviors in MGO-induced mice model, The OFT was performed in an open black box (60 × 60 × 60 cm). Mice were individually placed in the center of an open box and observed for 5 min 35. The total distance traveled was analyzed using the SMART v3.0, video tracking system (Panlab; Harvard Apparatus, Barcelona, Spain).

### Tail suspension test (TST)

To find the improvement in the depression by treating TP-41 in MGO-induced mice model, TST was performed in a TST chamber (60 cm length, 60 cm height, 11.5 cm depth, and 15 cm width), and each mouse was suspended using painless tape fixation. Before recording, all mice were acclimated to the TST chamber for 2 min. Subsequently, the immobility time of each mouse was recorded for 4 min 35 and analyzed using the SMART v3.0 video tracking system.

### Forced swim test (FST)

To investigate the anti-depression effect of TP-41 in MGO-induced mice model, FST was performed in an FST chamber (50 cm height × 20 cm diameter) filled with water (up to 30 cm) at room temperature (RT). Before recording, all mice were adapted to the FST chamber for 2 min. The time spent immobile for each mouse was measured in the subsequent 4 mins 36. The immobility time was analyzed with the SMART v3.0 video tracking system.

### Novel object recognition test (NORT)

To evaluate the spatial memory in the MGO-induced mice model by treating TP-41, NORT was performed in an open black box (60 cm × 60 cm × 60 cm). On the training day, the mice were placed in an open box with two different kinds of identical objects for 3 min 37. The next day, the mice were placed in the same box, in which one of the identical objects had been replaced with a novel object. The percentage of recognition (%) was calculated as (time spent exploring the novel object) / (total time spent exploring both objects) × 100. The NORT results, including the total distance traveled, were analyzed using the SMART v3.0, video tracking system.

### Y-maze test

Working memory was evaluated in a Y-shaped maze composed of three arms (30 cm × 5 cm × 15 cm) placed 120° apart 38. At the start of each session, mice were released from the end of one arm and allowed to explore the maze for 8 min. An alternation was scored when the animal entered all three arms consecutively without revisiting a previously chosen arm. The alternation (%) was calculated as (number of alteration) / (total arm entries - 2) × 100. An alternation was defined as entries into all three arms consecutively (*e.g.*, ABC, BCA, or CAB). Behavioral parameters, including arm entries and alternation percentage, were quantified using the SMART 3.0 SUPER PACK system.

### Barnes maze test (BM)

The Barnes maze apparatus was constructed from polyethylene and consisted of a circular platform (45 cm in diameter) with 20 equally spaced holes (4.5 cm in diameter) arranged around its perimeter 2. The platform was elevated 50 cm above the floor, and an escape box (35 × 25 × 15 cm) was positioned beneath one designated target hole. Each mouse was placed in the central starting zone and trained to locate the escape box over four consecutive days. The mean latency to reach the target hole was recorded and analyzed using SMART 3.0 SUPER PACK software.

### Enzyme-linked immunosorbent assay (ELISA) analysis

The levels of pro-inflammatory cytokines (interleukin-1β [IL-1β], interleukin-6 [IL-6], and tumor necrosis factor alpha [TNF-α]) and cortisol from mouse serum and lysate brain measured with ELISA kits (R&D Systems, Minneapolis, MN, USA). Those assays were quantified using colorimetric or quantification R&D systems assay kits according to the manufacturer's instructions.

### Free form levels of MGO in serum analysis

MGO levels were analyzed as previously described, with slight modifications 39. Briefly, each serum sample was incubated with 0.45 N perchloric acid for one day, and then reacted with 10 mM o-phenylenediamine (*o*-PD) for one day at RT. The reacted samples were centrifuged at 12,000 rpm for 30 min. The supernatant was filtered using 0.2 μm filters (Whatman, Dassel, Germany) and injected into a high-performance liquid chromatography system (Waters Corporation, Milford, MA, USA) equipped with a photodiode array detector (315 nm). Injected samples (10 µL) were processed with a constant flow rate (1.0 mL/min). The sample was analyzed using a Kromasil C^18^ column (250 mm × 4.6 mm, 5 µm) with 20% acetonitrile to induce an isocratic condition.

### Immunohistochemistry (IHC) for GR

Glucocorticoid receptor (GR) expression in the paraventricular nucleus (PVN; bregma -0.70 to -0.90 mm) was assessed by IHC. Brain sections were rinsed in PBS, incubated in 1% hydrogen peroxide for 15 min, and blocked in PBS (pH 7.4) containing 3% normal goat serum and 1% BSA. Sections were incubated overnight at 4 °C with mouse anti-GR antibody (1:500; Santa Cruz Biotechnology) in 0.3% Triton X-100. After PBS washes, sections were incubated with biotinylated anti-goat IgG (1:500) for 1 h, followed by the avidin-biotin complex (ABC, 1:100; Vector Laboratories) for 1 h at room temperature. Immunoreactivity was visualized with 3,3'-diaminobenzidine (DAB) in Tris-buffered saline (pH 7.6). Slides were mounted with DPX medium, and images were obtained at 100× magnification using an Olympus BX51 microscope.

### IHC for amyloid precursor protein (APP) and Aβ

Brains were fixed in 10% neutral-buffered formalin for 24h at 4 °C, dehydrated in graded ethanol, cleared in xylene, embedded in paraffin, and sagittally sectioned at 4 µm. After deparaffinization, sections were incubated overnight at 4 °C with antibodies against APP (1:200; Invitrogen, cat. 14974982), Aβ_1-42_ monomer (mAβ_1-42_, 1:200; Invitrogen, MA5-36246), and oligomeric Aβ (oAβ, 1:200; Invitrogen, AHB0052). Detection was performed using the avidin-biotin horseradish peroxidase complex (Vector Laboratories), and immunoreactivity was visualized with DAB. Images were captured at 100× magnification with a Nikon Eclipse 80i microscope.

### Confocal immunofluorescence

Paraffin-embedded brain sections (4 µm) were incubated with the primary antibody against Aβ (6E10, mouse, 1:500; Biolegend) and Iba1 (rabbit, 1:500; Wako) overnight at 4 °C. After washing, the sections were incubated with donkey anti-mouse Alexa Fluor 555 (1:500; Invitrogen, A-31570, RRID:AB_2536190) for Aβ, and donkey anti-rabbit Alexa Fluor 488 (1:500; Invitrogen, A-21206, RRID:AB_2535792) for Iba-1. Nuclei were counterstained with DAPI. Images were acquired using a Nikon A1+ laser scanning confocal microscope and analyzed with NIS-Elements software.

### Western blotting assay

Whole brains collected from mice were lysed in PRO-PREP protein extraction solution (iNtRON, Seoul, Korea) at -20°C for 24 h. Tissue lysates were separated by centrifugation at 12,000 rpm for 30 min, and protein content was determined using the Bradford assay. The Bradford assay was performed a colorimetric change of at 595 nm in microplate reader by using bovine serum albumin as standard. Proteins (30-50 µg) were separated by sodium dodecyl sulfate-polyacrylamide gel electrophoresis and transferred to polyvinylidene fluoride membranes. Membranes were incubated with primary antibodies, receptors of advanced glycation end products (RAGE) (1:500, Santa Cruz Biotechnology Inc., Santa Cruz, California, USA, cat. sc365154), APP (1:1000, Invitrogen, Carlsbad, CA, USA, cat. 14974982), mAβ_1-42_ (1:1000, Invitrogen, cat. MA5-36246), oAβ (1:1000, Invitrogen, Carlsbad, CA, USA, cat. AHB0052), tau (total form, 1:1000, Abcam, Cambridge, MA, USA, cat. Ab109390), phosphorylated-tau including monomer and oligomer types (1:1000, Abcam, cat. Ab254256), and α-tubulin (1:1000, Invitrogen, cat. A11126), at 4 °C for 1 d. Following incubation, the membranes were washed and incubated with secondary antibodies at RT for 2 h. Protein bands were measured using a ChemiDoc™ XRS+ imaging system (Bio-Rad Laboratories, Hercules, CA, USA).

### Statistical analysis

Statistical analyses were performed using one-way ANOVA followed by Tukey's post hoc test for multiple comparisons, except for the NORT which was analyzed using two-way ANOVA (treatment × object) with Tukey's post hoc test. Data are expressed as the mean ± SEM, and differences were considered statistically significant at *P* < 0.05. Animals were randomly assigned to treatment groups, and behavioral scoring and image quantification were performed in a blinded manner.

## Results

**Figure 1:**
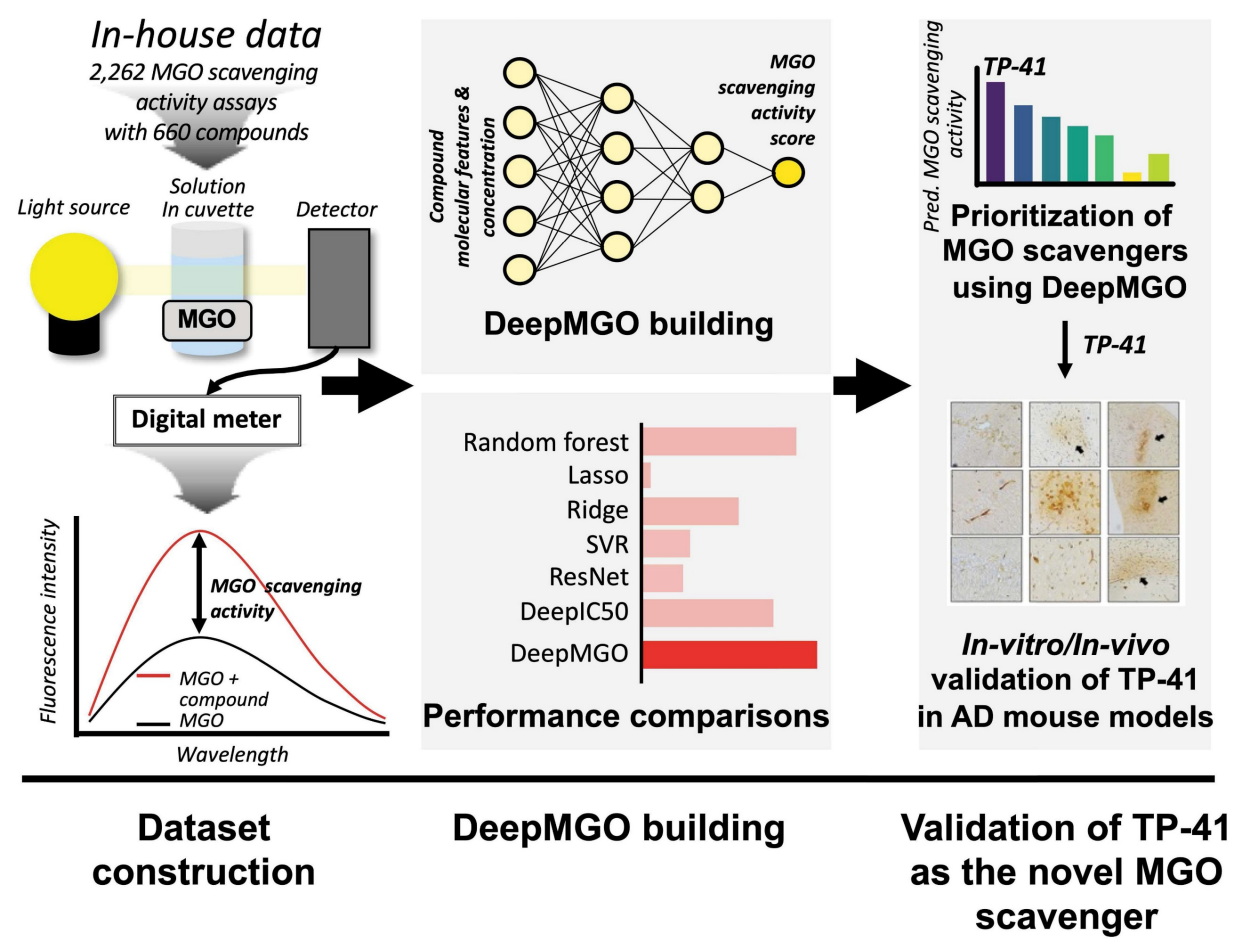
** Study design.** Using deep learning, our aim is to screen compounds (MGO scavengers) that bind to MGO with high affinity, finally scavenging MGO. Thus, we constructed a DL model for predicting MGO scavenging activity scores by scavenger compounds. First, we performed an MGO scavenging activity assay using in-house compounds to generate our own screening dataset for training an MGO scavenging activity prediction DL model, DeepMGO. We designed DeepMGO taking the molecular features and concentration of a compound as input, in order to obtain the MGO scavenging activity score of the compound. In addition, we compared the performance of DeepMGO with those of other DL models regarding MGO scavenging activity prediction of scavenger compounds. We also applied DeepMGO to identify a novel MGO scavenger from a compound library and inspected its therapeutic effect on AD by *in vitro* and *in vivo* experiments.

Using data from 2,262 MGO scavenging activity assays with 660 diverse compounds, including natural compounds, FDA-approved drugs, and amino acids, DeepMGO predicts the MGO scavenging activity scores of compounds by considering their molecular features and concentrations as inputs (Figure 1). Molecular features are represented as vectors with binary values (*i.e.*, presence or absence) of specific submolecular structures and continuous values, including molecular weight, polarizability, acidity, solubility, atom counts, and topological indices (Figure S1). We also compared the performance of DeepMGO with those of other DL and ML models (Figure 1) to confirm the utility of DeepMGO for the discovery of novel MGO scavengers.

### Structure and training of DeepMGO

**Figure 2:**
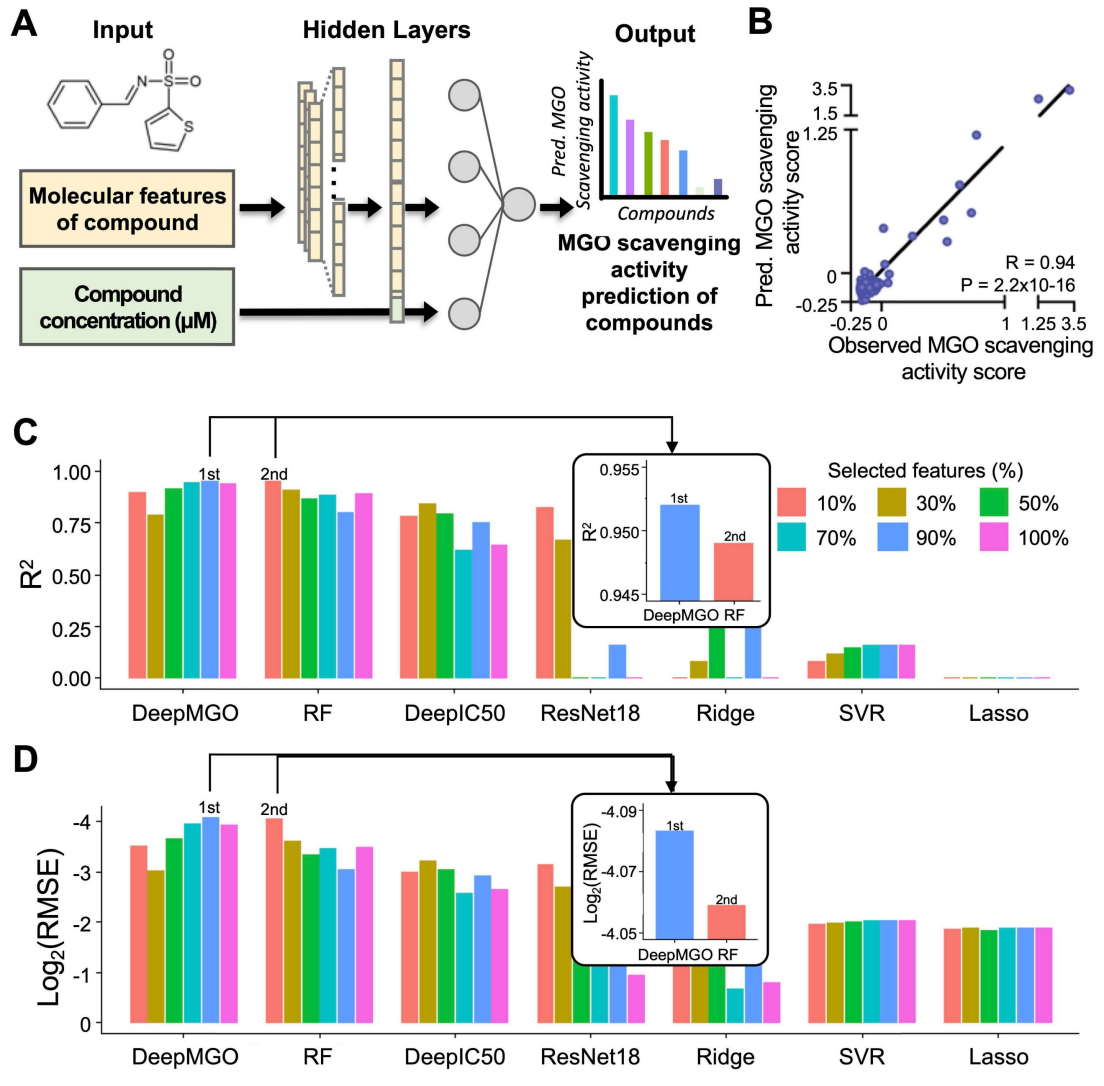
** Construction of DeepMGO and model performance comparison.** (A) DeepMGO takes the molecular features of a compound and its concentration (μM) as inputs to predict an MGO scavenging activity score. (B) Pearson's correlation coefficient was calculated between the observed MGO scavenging activity score in the test set and the predicted MGO scavenging activity score by DeepMGO using the test set. (C) R^2^ and (D) log_2_(RMSE) of MGO scavenging activity prediction models using selected features by feature selection (10, 30, 50, 70, 90%) and all features (100%). The model with a higher R^2^ and lower log_2_(RMSE) value is considered to have good performance. R^2^ with a 0.0 value includes the negative or zero R^2^ values. We labeled the model with the highest performance as '1st' and the model with the second-best performance as '2nd.'

To construct DeepMGO, we designed its architecture to consist of three convolutional and three dense layers (Figure 2A and Figure S2). The DeepMGO model was constructed using training and validation datasets with a full set of features, and its performance was subsequently evaluated using the test dataset. In subsequent experiments, feature selection was applied to optimize the performance of the model. However, the results in this section are based on a full set of features. For evaluation of DeepMGO in the test dataset, the PCC was calculated between the observed values in the test dataset and the values predicted by DeepMGO, resulting in a coefficient of 0.94, with a P value of 2.2 x 10^-16^ (Figure 2B). The performance of DeepMGO was compared to other models by determining R^2^ and log_2_(RMSE) values, wherein a higher R^2^ and lower log_2_(RMSE) indicate better performance. The R^2^ value for DeepMGO was 0.939, and the log_2_(RMSE) was -3.921 (pink bars in Figure 2C—D, Tables S6—S7).

### DeepMGO showed competitive performance compared to other DL and ML models

To compare the MGO scavenging activity prediction performance of DeepMGO, we constructed DL models using the architectures of DeepIC50 29 and ResNet18 30. For ML models, we utilized lasso, ridge, RF, and SVR to construct four prediction models with hyperparameter optimization. We compared the performance of each of the seven constructed models using a test dataset. In the ML models, lasso had an R^2^ of -0.026 and log_2_(RMSE) of -1.878; ridge had an R^2^ of -3.53, log_2_(RMSE) of -0.808; RF had an R^2^ of 0.889 and log_2_(RMSE) of -3.474; and SVR had an R^2^ of 0.158 and log_2_(RMSE) of -2.023. In the DL models, ResNet18 had an R^2^ of -2.754 and a log_2_(RMSE) of -0.943, whereas DeepIC50 had an R^2^ of 0.640 and a log_2_(RMSE) of -2.635 (Figure 2C—D, Tables S6—S7). Collectively, DeepMGO exhibited the best performance compared with the other models.

### Feature selection for DL and ML models

Furthermore, we aimed to verify whether the performance of the MGO scavenging activity prediction model was improved through feature selection by reducing overfitting. To achieve this, the feature importance was evaluated using univariate linear regression tests. Subsequently, the 10, 30, 50, 70, and 90% important features, among all the features in the training dataset were selected to construct the ML and DL models. The performance of each model was evaluated using a test dataset (Figure S1).

DeepMGO performed superiorly (at 90% selected features) compared to other models (refer to the inset bar plots in Figure 2C—D, Figure S5, Tables S6—S7). The R^2^ values of DeepMGO ranged from 0.790 to 0.952, with log_2_(RMSE) values ranging from -4.083 to -3.023. The DeepIC50 values R^2^ ranged from 0.615 to 0.842 and log_2_(RMSE) ranged from -3.224 to -2.582, while R^2^ of ResNet18 ranged between -2.754 to 0.821 and log_2_(RMSE) from -3.133 to -0.943. The R^2^ of the lasso ranged from -0.091 to -0.026, and the log_2_(RMSE) ranged from -1.878 to -1.837. The R^2^ of the ridge ranged from -4.403 to 0.667, and log_2_(RMSE) ranged from -2.690 to -0.680. R^2^ of SVR ranged from 0.082 to 0.158, with log_2_(RMSE) values from -2.023 to -1.960. The R^2^ of the RF ranged from 0.797 to 0.949, and the log_2_(RMSE) ranged from -4.059 to -3.047 (Figure 2C—D, Tables S6—S7). Among the ML models, the RF models showed the best performance but did not surpass DeepMGO. Overall, DeepMGO consistently outperformed the other models, regardless of feature subset size, and reached optimal results at 90% feature selection, which was adopted as the final model (DeepMGO).

### Evaluation of DeepMGO using an independent test dataset

**Figure 3:**
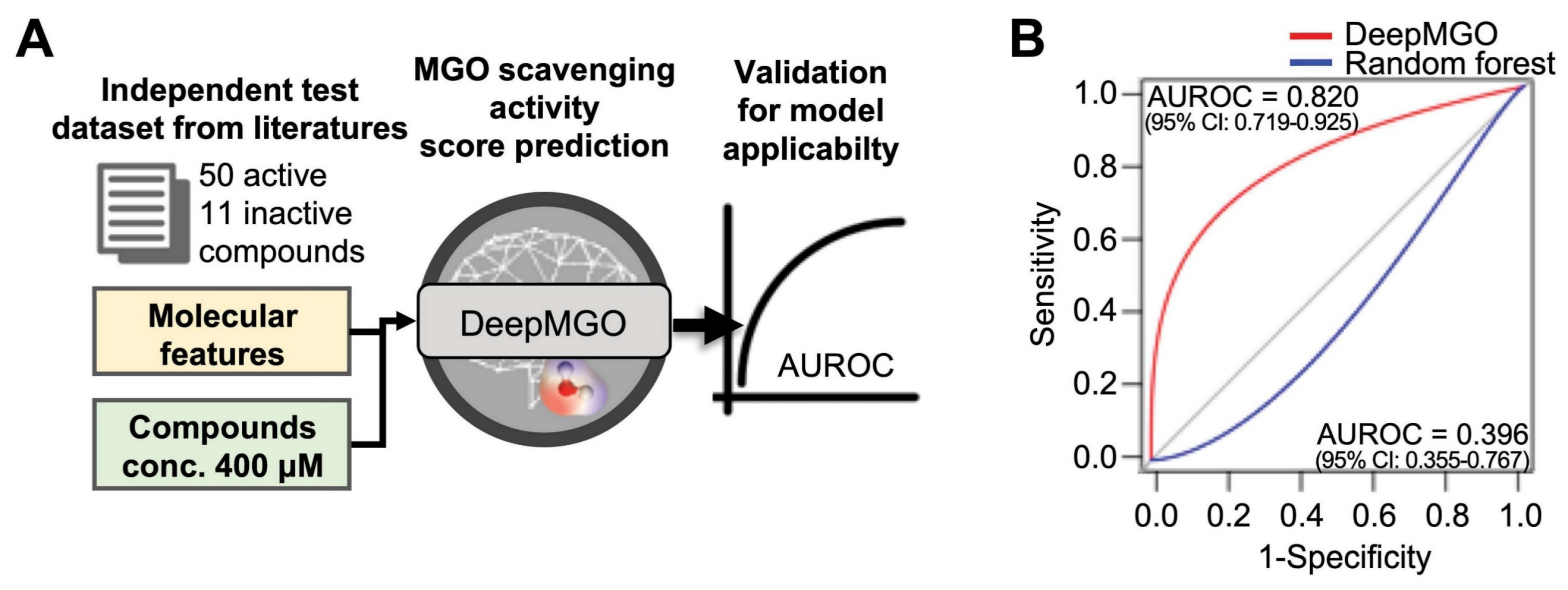
** Validation for applicability of the DeepMGO model using an independent dataset.** (A) Scheme of validation for model applicability using an independent dataset. (B) AUROC curve of DeepMGO (with 90% selected features) and random forest (with 10% selected features).

To validate the performance of DeepMGO with an independent test dataset, we curated 61 MGO scavengers (50 active and 11 inactive to MGO) from literature sources as an independent test dataset (Figure 3A and Table S5) 40-64. MGO scavenging activity prediction scores were determined for 61 compounds for each MGO scavenging activity prediction model (Figure 3A). The active state of the investigated external compounds was assigned to a value of 1, whereas the inactive state was assigned to a value of 0. The AUROC was calculated for evaluation.

The AUROC for DeepMGO was 0.820 (95% confidence interval [CI]: 0.719-0.925). AUROC of the RF model with 10% features had the second greatest value at 0.396 (95% CI: 0.355-0.767; blue line in Figure 3B). Compounds were predicted to be active in DeepMGO when the MGO scavenging activity score was greater than -0.186, as determined by the AUROC value.

Taken together, we selected DeepMGO using 90% features as the MGO scavenging activity prediction model for screening novel MGO scavengers.

### TP-41 was prioritized by DeepMGO

**Figure 4:**
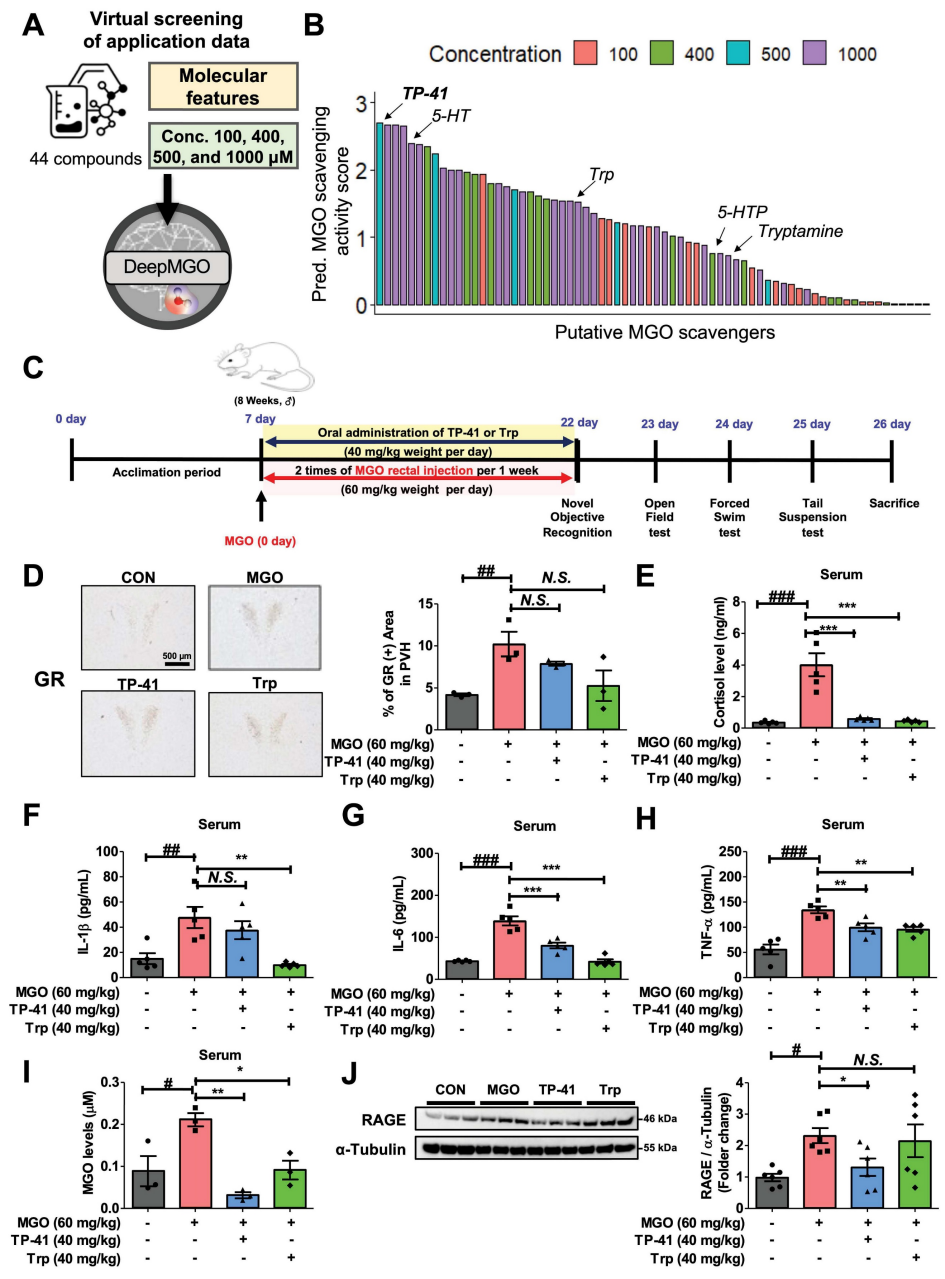
** Identification and validation of TP-41 as a novel MGO scavenger and putative therapeutic compound for AD.** (A) Scheme of virtual screening of 44 compounds in the application data for prioritization and identification of a novel MGO scavenger. (B) Among the 44 compounds, the predicted MGO scavenging activity score of TP-41 was the highest. (C) Schematic diagram of the experimental plan for the MGO-induced depression and memory loss mouse model. (D) Immunostaining assays were performed to detect GR expression in the PVN. The GR-immunoreactive cells were quantified by GR-positive cells in the PVN. Scale bar = 500 µm. (E) Densitometry graph of serum cortisol levels measured using ELISA. (F-H) Densitometry graph of pro-inflammatory cytokine levels for IL-1β (F), IL-6 (G), and TNF-α (H) in serum was measured using ELISA. (I) Densitometry graph of detected MGO levels in serum analyzed using high-performance liquid chromatography. (J) The protein expression level of RAGE in mouse brains was analyzed by western blotting. Quantitative analysis of RAGE expression was normalized using α-tubulin as the loading control. Values are represented as mean ± SEM (N = 3-6). ^#^*P* < 0.05, ^##^*P* < 0.01, and ^###^*P* < 0.001 vs. control group (CON). ^*^P < 0.05, ^**^P < 0.01, and ^***^P < 0.001 vs. MGO group (MGO). GR: glucocorticoid receptor; PVN: paraventricular nucleus of hypothalamus.

We subsequently utilized the predictive capabilities of DeepMGO (90% features) for MGO scavenging activity to identify novel MGO scavengers as potential AD therapeutics. Forty novel compounds derived from Trp with the greatest affinity for MGO and four putative MGO scavenger candidates, along with reference 5-HT, Trp, tryptamine, and 5-HTP, were prepared to compare their MGO-scavenging effects (Figure 4A and Figure S3). Each compound was screened at concentrations of 100, 400, 500, and 1,000 μM, and the predicted MGO scavenging activity score was determined. The Trp-derived molecule TP-41 (500 μM) exhibited the highest predicted score (Figure 4B and Table S8).

To further assess TP-41's BBB permeability, we used tools logBB_Pred 31 and ADMET-AI 32, comparing the predicted BBB permeability of the reference compounds 5-HTP and 5-HT (also known to pass the BBB) 65, 66. The logBB_Pred model indicated that TP-41 (LogBB = -0.9758) was BBB permeable (using a cutoff of LogBB < -1 for non-permeable), similar to 5-HT (LogBB = -0.8031), while 5-HTP (LogBB = -1.1640) was predicted to be BBB non-permeable (Figure S6A). To further validate this finding, we used ADMET-AI, where the predicted BBB score was 0.7737 for TP-41, substantially higher than 5-HTP (0.6010) and 5-HT (0.5580) (Figure S6B), strongly supporting its suitability for in vivo studies targeting the brain. Therefore, we further investigated the therapeutic effects of TP-41 in an AD mouse model 2.

### TP-41 attenuates Hypothalamic-Pituitary-Adrenal (HPA) Axis Dysregulation and Neuroinflammation in MGO-induced mouse

Based on the DeepMGO results, we examined TP-41 as a promising MGO scavenger in an MGO-induced mouse model of depression and cognitive impairment (Figure 4C), focusing on HPA axis dysregulation and neuroinflammation. MGO administration increased the amounts of GR protein expression in the hypothalamic paraventricular nucleus (PVN) and cortisol levels in the serum (Figure 4D-E). To evaluate the effect of TP-41 in the MGO-induced mouse model, we administered TP-41 (40 mg/kg) to mice and compared it to mice treated with the antidepressant Trp (40 mg/kg) as a positive control (PC) (see Figure 4C). Remarkably, the treated TP-41 mice had reduced levels of GR in the PVN and cortisol in the serum, similar to the levels in the Trp-treated mice (Figure 4D-E).

**Figure 5:**
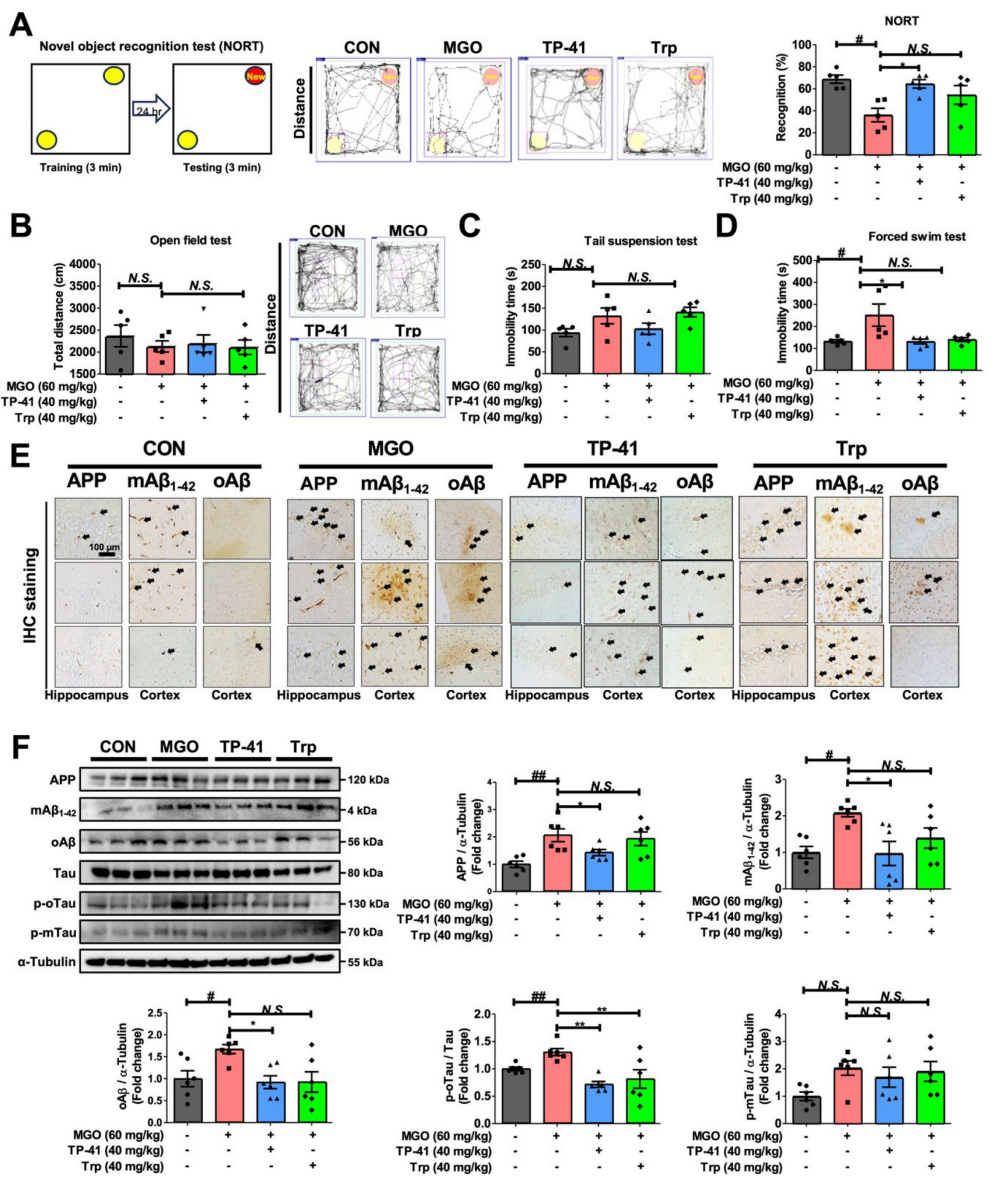
** Investigation of TP-41 administration in an MGO-induced depression and memory loss mouse model.** (A) In the NORT, the total distance and recognition percentage were recorded and analyzed. (B) In the OFT, the total distance was analyzed. (C-D) In TST and FST, the immobility times were analyzed. (E) Immunostaining assay was performed to detect APP, mAβ_1-42_, and oAβ expression in both the hippocampus and cortex. (F) The protein expression levels of APP, mAβ_1-42_, oAβ, Tau, p-oTau, and p-mTau in mouse brains were analyzed by western blotting. Quantitative western blot analysis of each protein included normalization with α-tubulin as a loading control. Values are represented as the mean ± SEM (N = 3-6). ^#^*P* < 0.05 and ^##^*P* < 0.01 vs. control group (CON). ^*^P < 0.05, ^**^P < 0.01, and ^***^P < 0.001 vs. MGO group (MGO).

Moreover, MGO administration increased the levels of cytokines (IL-1β, IL-6, and TNF-α) in the serum, which led to neuropsychiatric disorders and depression as shown in behavior tests (*i.e.*, OFT, TST, and FST) (Figure 4F-H and Figure 5B-D). Levels of the free form of MGO in the serum, and RAGE protein expression levels in the mouse brain, were significantly increased in MGO-treated mice, indicating that MGO might be considered a more potent biomarker for AD progression (Figure 4I-J).

In addition, MGO-treated mice also significantly increased pro-inflammatory cytokines (*i.e.*, IL-1β, IL-6, and TNF-α) and the free form of MGO in serum (Figure 4F-I). Conversely, TP-41 administration significantly reduced RAGE expression in the MGO-induced mouse model, suggesting that TP-41 may attenuate MGO-induced changes by modulating the RAGE pathway and consequently inhibiting NF-κB activation 67 (Figure 4J).

### TP-41 ameliorated the depression and memory loss in MGO-treated mice

To clarify whether MGO affects cognitive and depression-like behaviors in ICR mice, we performed NORT experiments to evaluate memory loss. The percentage of recognition after treatment with MGO was significantly lower than that in the control (CON) mice, implying that MGO induces memory dysfunction (Figure 5A).

Moreover, we conducted several depressive behavior tests (OFT, TST, and FST). The OFT was used to assess anxiety behavior, and the MGO-treated mice groups showed a slight reduction in the total distance traveled compared with the CON group (Figure 5B). The TST, another technique used to measure depression-like activity, demonstrated that MGO-induced a longer immobility time in mice than in the control group (Figure 5C). Notably, we found that MGO strongly triggered immobility in ICR mice in the FST. Collectively, all behavioral tests showed that MGO-induced depression/anxiety and memory loss in mice, possibly by increasing the levels of inflammatory factors, including cytokines and RAGE from serum and brain after sacrificing the mice (Figure 5D).

AD is characterized by the accumulation of Aβ and hyperphosphorylated tau protein in the mice brains 68. Thus, we verified Aβ and tau activation as major pathological features of AD in this novel *in vivo* model induced by MGO. MGO treatment in mice considerably increased the expression of APP, mAβ, and oAβ in comparison with CON mice, and also increased the expression of the phosphorylated-oligomeric form of tau (p-oTau; Figure 5E-F).

The expression levels of the phosphorylated monomeric form of tau (p-mTau) after treatment with MGO were slightly higher than those in the CON group (Figure 5F). Our results indicated that MGO could induce tau phosphorylation and Aβ deposition in the brains of mice.

We further analyzed the effects of TP-41 on cognitive impairment and depressive behavior in MGO-treated mice. In NORT, TP-41 rescued the ability of mice to recognize new objects in MGO-induced mice model (Figure 5A). In the OFT, which assesses locomotor/exploratory and anxiety-like behaviors, TP-41 slightly increased the total distance traveled compared to the MGO group (Figure 5B). The immobility time was reduced in the treated TP-41 mice group in the TST and FST, comparing with MGO-induced group (Figure 5C-D).

To elucidate the mechanisms underlying AD-related pathology, we evaluated the effect of TP-41 on MGO-activated Aβ types and tau phosphorylation using western blotting and IHC staining assays (Figure 5E-F). TP-41 strongly suppressed MGO-induced APP and Aβ type (monomer and oligomer) expression levels in both western blotting and IHC assays (Figure 5E-F). These results indicate that TP-41 protects mice from MGO-induced memory loss *via* its MGO-scavenging capacity, which further regulates RAGE expression, inflammatory factors, and HPA axis-related markers.

In addition, TP-41 treatment significantly reduced the expression level of p-oTau compared to the MGO group (Figure 5F). Although p-mTau expression also showed a decreasing trend, the large inter-individual variability prevented statistical significance. These findings suggest that TP-41 contributes to the suppression of tau hyperphosphorylation, particularly p-oTau, which is closely associated with synaptic impairment and neuronal degeneration, thereby alleviating neuronal dysfunction 69.

Depression is typically associated with short-term memory problems. Dementia, including AD and depression, are closely associated. Previous reports have suggested that depression can also be a symptom of AD, particularly in the early stages. Therefore, TP-41 identified by DeepMGO may be a novel therapeutic compound for the early stages of AD.

### TP-41 ameliorates memory deficits and modulates amyloid and tau in 5xFAD mice

**Figure 6:**
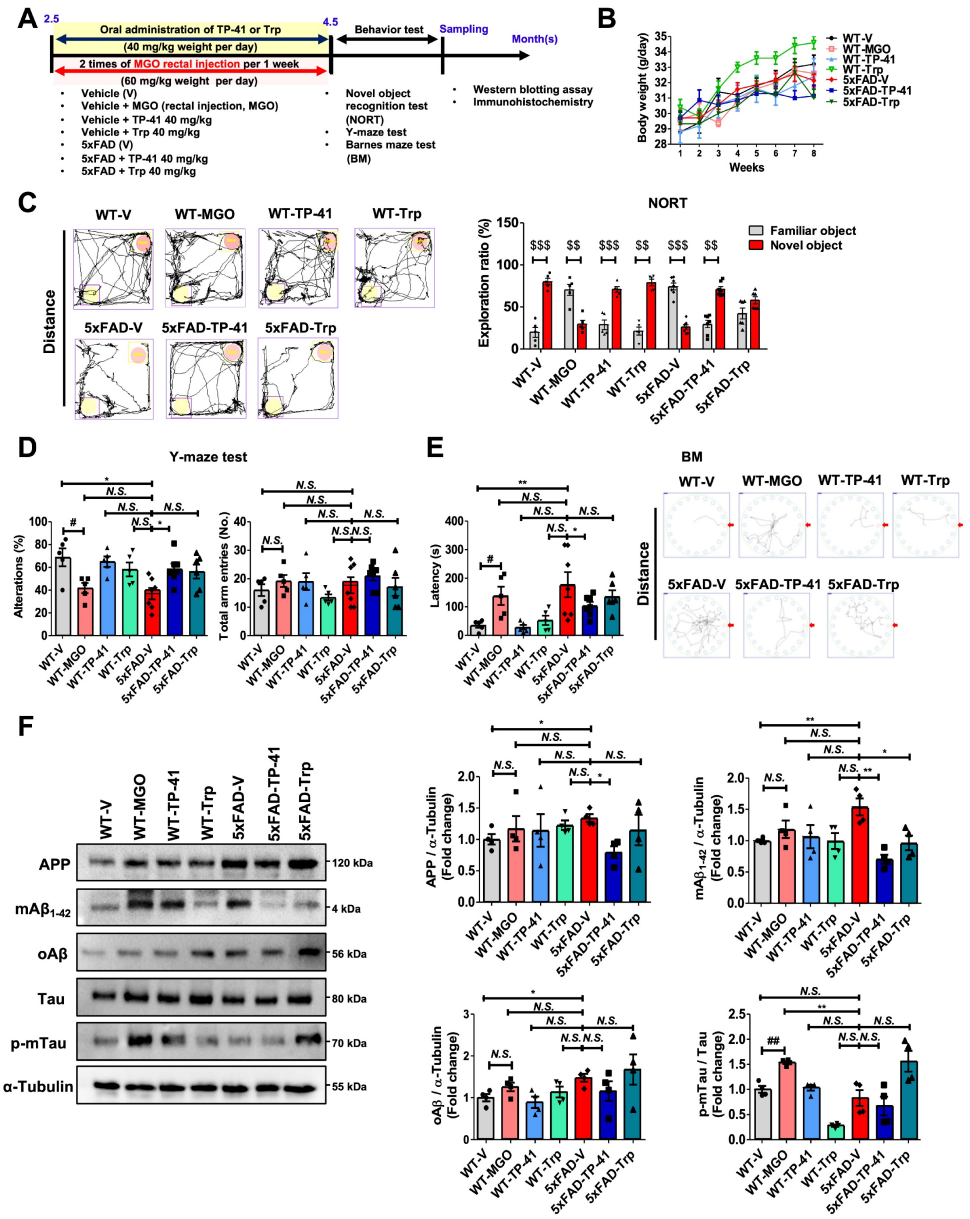
** TP-41 ameliorates memory deficits and modulates amyloid and tau in 5xFAD mice.** (A) Schematic diagram of experimental design. (B) Body weight changes during the experimental period. (C) In the novel object recognition test (NORT), the exploration ratio (%) was analyzed. ^$$^*P* < 0.01 and ^$$$^*P* < 0.001 vs. familiar object group (two-way ANOVA). (D) In the Y-maze test, the percentage of spontaneous alternation and total entries were measured. (E) In the Barnes maze (BM), escape latency and total distance were recorded. (F) The protein expression levels of APP, mAβ_1-42_, oAβ, and Tau, and p-mTau in mouse brains were analyzed by western blotting. Quantitative western blot analysis of each protein included normalization with α-tubulin as a loading control. Values are represented as the mean ± SEM (N = 4-7). ^#^*P* < 0.05 and ^##^*P* < 0.01 vs. WT control (WT-V). ^*^P < 0.05 and ^**^P < 0.01 vs. 5xFAD-vehicle (5xFAD-V).

To enhance translational relevance, we examined the effects of TP-41 in the 5xFAD transgenic mouse model of AD, which recapitulates amyloid and tau pathology (Figure 6A). Oral administration of TP-41 (40 mg/kg) improved depressive-like behaviors in 5xFAD mice without affecting body weight (Figure 6B). Cognitive function was evaluated using the NORT, Y-maze, and Barnes maze. In the NORT, 5xFAD mice showed a significantly lower exploration ratio compared with WT controls, indicating impaired recognition memory. MGO-induced WT mice also exhibited poor exploration similar to 5xFAD mice. TP-41 treatment improved recognition performance, whereas Trp supplementation had only a modest effect (Figure 6C). In the Y-maze, both 5xFAD and MGO-induced WT mice displayed reduced alternation behavior, reflecting working memory deficits, suggesting that MGO exposure contributes to memory dysfunction. TP-41 significantly increased spontaneous alternation compared with vehicle-treated 5xFAD mice, while Trp supplementation was less effective (Figure 6D). In the BM, 5xFAD and MGO-induced WT mice exhibited prolonged escape latencies and impaired spatial learning. TP-41-treated 5xFAD mice showed shorter escape latencies and improved probe trial performance, whereas Trp treatment provided only partial improvement (Figure 6E).

At the molecular level, Western blot analysis revealed increased APP, mAβ_1-42_, oAβ, and phosphorylated tau in 5xFAD mice compared with WT controls. Notably, MGO-induced WT mice showed a pronounced increase in p-mTau expression, suggesting that MGO primarily affects tau-related rather than amyloid-related pathways. TP-41 reduced APP and Aβ accumulation, and tau phosphorylation, while Trp exerted only minor effects (Figure 6F). These findings demonstrate that TP-41 improves recognition, working, and spatial memory in 5xFAD mice and alleviates amyloid and tau pathology, while also underscoring the contribution of MGO to tau-associated neurotoxicity.

### TP-41 reduces amyloid deposition and neuroinflammatory responses in the hippocampus and cortex of 5xFAD mice

**Figure 7:**
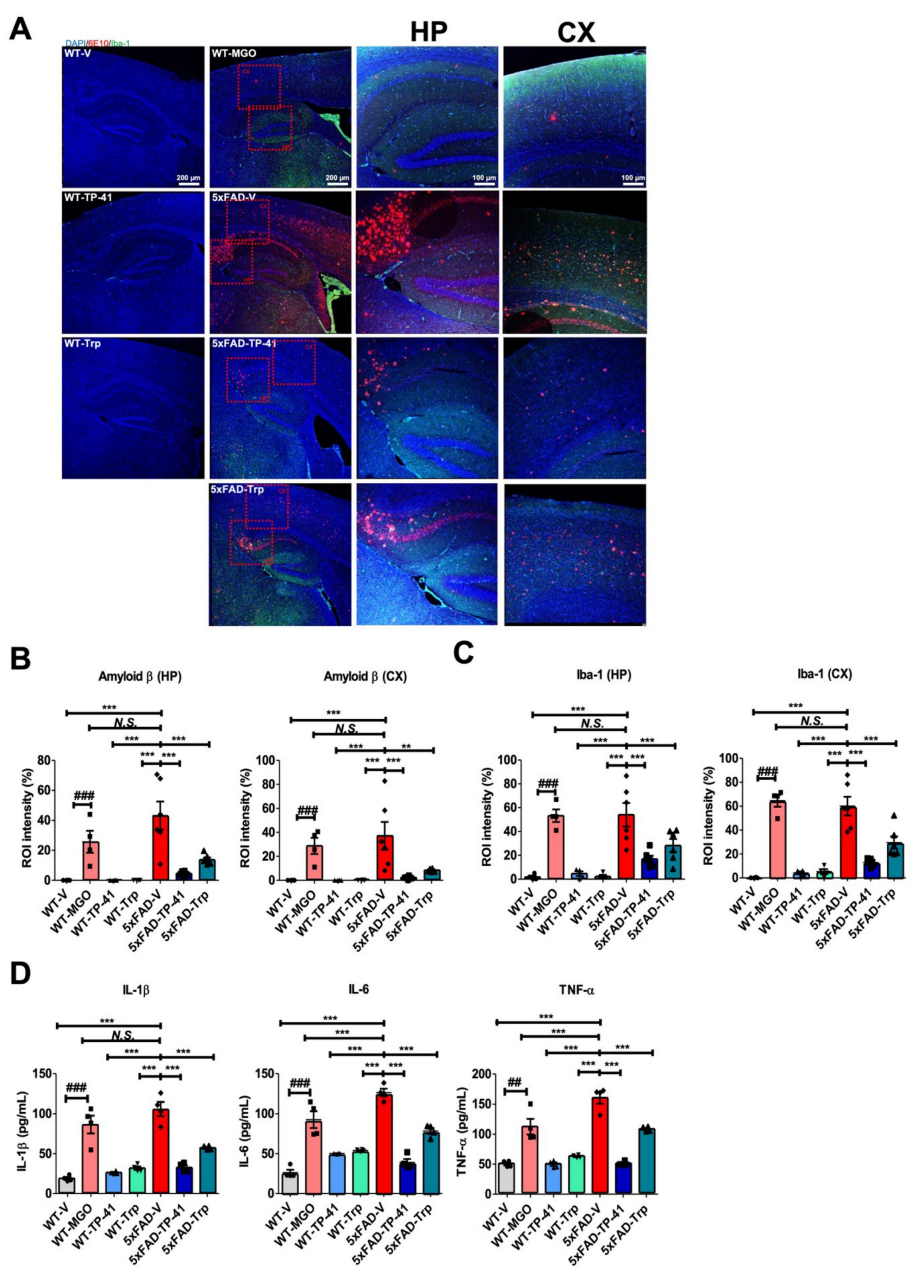
** TP-41 reduces amyloid deposition and neuroinflammatory responses in the hippocampus and cortex of 5xFAD mice.** (A-C) Immunofluorescence staining was performed to detect Aβ (6E10) and Iba-1 expression in the HP and CX. Representative images show Aβ (red) and Iba-1 (green) immunofluorescent signals. Scale bar: 100 μm (10× magnification) - 200 μm (4× magnification). Quantitative analysis of fluorescence intensity in HP and CX regions was expressed as region of interest (ROI) intensity ratios (%). (D) Cytokine levels of IL-1β, IL-6, and TNF-α, in whole brain lysates were measured. Values are represented as the mean ± SEM (N = 4-6). ^###^*P* < 0.001 vs. WT control (WT-V). ^**^P < 0.01 and ^***^P < 0.001 vs. 5xFAD-vehicle (5xFAD-V).

Amyloid deposition and neuroinflammation were assessed in the hippocampus (HP) and cortex (CX). Immunofluorescence revealed minimal Aβ (6E10) and Iba-1 signals in WT controls, whereas both MGO-induced WT and 5xFAD mice showed marked amyloid accumulation and microglial activation (Figure 7A-C). Transgenic mice also exhibited strong Aβ expression. Quantitative analysis confirmed significant increases in amyloid-β and Iba-1 compared with WT controls.

TP-41 treatment markedly reduced amyloid-β deposition and Iba-1-positive microglial activation in both HP and CX of 5xFAD mice, while Trp supplementation showed only partial effects (Figure 7A-C). Consistently, cytokine profiling of brain lysates revealed elevated TNF-α, IL-1β, and IL-6 in MGO-induced WT and 5xFAD groups, which were significantly attenuated by TP-41 but only modestly reduced by Trp (Figure 7D). These findings indicate that TP-41 reduces amyloid burden and neuroinflammatory responses in both hippocampal and cortical regions, accompanied by suppression of pro-inflammatory cytokine production.

## Discussion

Increasing evidence indicates that MGO, a glycolytic byproduct, plays a critical role in AD pathogenesis 2. Clinical studies have linked hyperglycemia to depression and memory loss in diabetic patients, while experimental models show that MGO accelerates brain damage, cognitive decline, and AD-like pathology 70, 71. AGEs, which are closely associated with AD, promote oxidative stress and neuroinflammation, driving neurodegeneration 72. As a major precursor of AGEs, MGO represents a critical pathogenic factor in AD, beyond its role in AGE formation. Therefore, targeting MGO offers a promising therapeutic strategy to counteract cognitive dysfunction. To this end, we developed DeepMGO, a novel DL-based platform designed to identify candidate MGO scavengers and establish MGO as a tractable therapeutic pathway in AD (Figures 1-2).

DeepMGO's superior performance arises from its parsimonious architecture, comprising only 0.22 million parameters, which is particularly well-suited for our high-dimensional biochemical assay dataset (p > n; the number of features, 2,756, exceeds the number of samples, 2,262), a situation commonly encountered in quantitative structure-activity relationship (QSAR) studies 73, 74. High-dimensional datasets are inherently prone to the curse of dimensionality, making them susceptible to overfitting, especially when models possess excessive capacity. Indeed, complex models such as DeepIC50 (32.45 million parameters) and ResNet18 (3.88 million parameters) were observed to learn noise rather than meaningful patterns 75.

This was evident in our feature selection experiments—a standard diagnostic for overfitting in QSAR modeling 74—where ResNet18's performance dramatically improved, with its R² value increasing from -2.754 to 0.821 when using only 10% of features compared to all features (Figure 2C-D, Tables S6-S7). In contrast, DeepMGO's streamlined design functions as implicit structural regularization, meaning that the architecture itself constrains model complexity and prevents overfitting, enabling it to capture true underlying patterns and achieve stable, high performance (R² = 0.952 with 90% of features) without learning noise. While deeper architectures may be beneficial for larger datasets in the future, dimensionality reduction strategies such as autoencoders could also be considered 76.

To interpret the major features DeepMGO used over other models, an XAI using SHAP inspected which features contributed to the model performance (Figure S7). DeepMGO bases its predictions on a balanced set of fundamental physicochemical properties, including structural complexity based on valence paths (VP-1), polarity derived from atomic properties (BCUTp-1h), and molecular 3D shape (SpMin4_Bhm) (Table S9), demonstrating a chemically intuitive and robust learning approach. In contrast, DeepIC50 relies heavily on a single structural feature (nTDHeteroRing: hexagonal ring structures), indicating a simplistic, less generalizable strategy. ResNet18 showed near-zero feature contributions, indicating that it failed to extract meaningful patterns from the descriptors. This suggests the model relied on noise rather than signal, leading to poor predictive performance. Consequently, ResNet18 is inferior to DeepMGO in both interpretability and robustness.

Overall, these results demonstrate that DeepMGO provides a robust and generalizable framework for virtual screening, particularly in emerging pathogenic pathways like MGO in AD, where biochemical assay data for training deep learning models are limited.

DeepMGO analysis revealed that high-affinity indole derivatives, including TP-41, TP-20, and TP-3, commonly carry electron-donating substituents (-OH or -OCH_3_) at the 5-position of the indole ring and flexible amino group-containing side chains (Figure 4B and Figure S3). TP-41 showed the highest predicted affinity, likely due to its spermidine-conjugated structure providing multiple nucleophilic sites 25, 77. This structure-activity relationship This structure-activity relationship highlights the importance of both indole core modifications and amino group-containing side chains for optimizing MGO scavengers. Consistent with these predictions, TP-41 ameliorated depression-like behaviors and cognitive deficits in MGO-induced mice (Figures 3-5). These findings support MGO scavenging as a potential therapeutic strategy for AD and establish a DL-guided biochemical screening framework for accelerating MGO-targeted drug discovery 18-21, 78, 79.

In our model, MGO not only elevated depression-related markers such as GR and cortisol but also increased free MGO and pro-inflammatory cytokines (IL-1β, IL-6, TNF-α), implicating activation of inflammatory pathways that underlie psychoneuroimmunity-related depression. Consistently, MGO treatment markedly upregulated RAGE and downstream NF-κB signaling, further driving cytokine production (Figure 5F). Although TP-41 more effectively lowered serum MGO levels than Trp, reductions in IL-1β and IL-6 did not strictly mirror this effect, suggesting additional contributions from Trp-related metabolic pathways or tissue-specific inflammatory cascades. Collectively, these findings support MGO as a pathogenic driver of depressive behavior and memory loss, reinforcing our hypothesis that MGO can promote cognitive decline alongside depression and AD-like pathology in this mouse model.

At the molecular level, MGO markedly increased APP, mAβ_1-42_, and oAβ expression in the hippocampus and cortex. Since Aβ plaques are a pathological hallmark of AD 80, 81, our findings, together with previous data, indicate that MGO is closely linked to AD biomarkers, including APP, mAβ, tau, and oAβ 2. These results suggest that MGO accelerates Aβ aggregation and deposition, supporting glycotoxin regulation as a potential therapeutic strategy for AD. In addition, tau hyperphosphorylation, a key driver of synaptic dysfunction in oAβ-mediated pathology 82, was strongly induced by MGO, consistent with prior reports implicating RAGE and AGEs formation 69. Thus, targeting MGO may help prevent both Aβ accumulation and tau hyperphosphorylation in AD-like pathology.

An important finding is that TP-41, the top candidate identified by DeepMGO, effectively reduced AD-related pathology and cognitive dysfunction in the MGO-induced mouse model. Both TP-41 and Trp improved recognition memory in the NORT and restored locomotor activity and immobility times in the OFT, TST, and FST. At the molecular level, TP-41 significantly reduced p-oTau expression, suppressed GR, cortisol, and pro-inflammatory cytokines (IL-1β, IL-6, TNF-α), and decreased RAGE expression, suggesting attenuation of HPA axis hyperactivation and neuroinflammation. Notably, the anti-depressant-like effects of Trp are likely attributable to a dual mechanism, involving both MGO scavenging and its role as a serotonin precursor. In contrast, TP-41 achieved comparable efficacy primarily through direct glycotoxin neutralization, underscoring its potential as a more specific anti-glycotoxin therapeutic candidate. Furthermore, TP-41 significantly lowered serum MGO levels, supporting the concept that targeting glycotoxins represents a promising therapeutic approach for both depression and AD.

Building upon these findings, we validated TP-41 in the 5xFAD transgenic model, which recapitulates amyloid and tau pathology (Figures 6 and 7). TP-41 significantly improved recognition, working, and spatial memory in the NORT, Y-maze, and Barnes maze (Figures 6). At the molecular level, TP-41 decreased APP, mAβ, oAβ, and phosphorylated tau. Furthermore, it more effectively attenuated amyloid deposition, microglial activation, and pro-inflammatory cytokines (TNF-α, IL-1β, IL-6) than Trp (Figure 7). Collectively, these results demonstrate that TP-41 exerts multimodal therapeutic actions by improving cognition and suppressing amyloid/tau pathology and neuroinflammation in AD models.

Interestingly, exogenous MGO administration in WT mice produced severe cognitive deficits than those observed in 5xFAD mice (Figures 6 and 7). Yet, immunohistochemistry showed only minimal Aβ deposition in the hippocampus and cortex, suggesting that these impairments were not primarily amyloid-driven. Instead, MGO markedly increased pro-inflammatory cytokines and microglial activation, indicating that glycotoxin stress induces neuropsychiatric and cognitive symptoms predominantly through neuroinflammatory pathways. These results highlight MGO as a distinct pathogenic factor linking metabolic stress to mood and cognitive dysfunction, complementing the amyloid-centric pathology of AD.

RAGE has been reported to bind Aβ, with receptor expression elevated in AD patients 83. Consistent with this, MGO increased APP, mAβ_1-42_, and oAβ in the hippocampus and cortex, which were attenuated by TP-41 or Trp (Figure 5E). Notably, TP-41 also reduced p-oTau levels, indicating its ability to inhibit MGO-induced tau hyperphosphorylation and thereby mitigate oAβ-mediated synaptic dysfunction 82.

Despite these promising results, several mechanistic questions remain. Although TP-41 demonstrated *in vivo* MGO scavenging activity, its direct molecular interaction with MGO should be clarified using structural biology. The precise signaling cascade linking MGO scavenging to behavioral improvements also remains unresolved, and potential off-target pathways require evaluation through selective inhibition studies. In addition, structure-activity relationship analyses will be necessary to define the pharmacophores of TP-41 and guide further optimization. Finally, the small sample size (n = 4-7 per group) limits statistical power, underscoring the need for larger studies to confirm the robustness and reproducibility of these findings.

Although the MGO dose used here (60 mg/kg) exceeds physiological levels, it was selected to induce reproducible pathology and enable mechanistic interrogation of glycotoxin-driven neurodegeneration. LC analysis (Figure S8) confirmed that TP-41 and Trp directly quenched free-form MGO, validating their specific mode of action. To enhance translational relevance, we further tested TP-41 in the 5xFAD model, where it significantly ameliorated behavioral deficits, amyloid/tau pathology, and neuroinflammation. These findings support TP-41 as an effective MGO scavenger with disease-relevant efficacy. Nonetheless, future studies employing lower-dose, chronic MGO exposure or metabolic/diet-induced models are required to approximate physiological conditions and exclude potential off-target effects. Beyond MGO, other aldehydes such as 4-hydroxynonenal and formaldehyde also contribute to AD pathology, and extending the DeepMGO framework to identify their scavengers could broaden therapeutic scope. Importantly, MGO holds unique pathogenic relevance as both a direct cytotoxin and a major precursor of AGEs, linking it to oxidative stress, neuroinflammation, and tau/Aβ pathology. Moreover, its depletion is closely tied to Trp metabolism and depression-like phenotypes, highlighting its dual role in neuropsychiatric and neurodegenerative processes.

In this study, a fluorescence-based assay was used as a rapid screening tool to assess compound reactivity toward MGO, and its predictive value was validated by LC analysis (Figure S8). We recognize that this assay is optimized for detecting carboline-type products arising from Trp-MGO interactions, but it does not fully capture non-fluorescent imine-forming scavengers such as carnosine. To address this limitation, we performed chemical profiling of the fluorescence readout products based on LC-qTOF-MS analysis (Figure S4). In this experiment, we confirmed that TP-41 and Trp directly quenched free-form MGO *via* Pictet-Spengler reaction and spontaneous dehydrogenation, suggesting a plausible molecular mechanism of MGO scavenging activity of TP-41 and Trp. This combined approach strengthens confidence in our findings and underscores the need to consider both fluorescent and non-fluorescent pathways when evaluating anti-glycotoxin activity.

Indole-derived natural products and analogues showed similar effects, further supporting the assay's utility. Consistent with our previous findings 2, Trp exhibited the highest reactivity among amino acids and was depleted under high-dose MGO exposure, contributing to neuropsychiatric impairments. Supplementation restored these deficits, underscoring the biological significance of stoichiometric scavenging. Although such mechanisms may limit large-scale clinical translation, they remain useful for identifying and ranking anti-glycotoxin scaffolds. Importantly, TP-41 also demonstrated efficacy in the 5xFAD transgenic model, extending its therapeutic potential beyond the artificial MGO-induced system to genetically driven amyloid and tau pathology.

Given that MGO and its downstream effectors (*e.g*., RAGE, phosphorylated tau) were consistently modulated in our study, these molecules may also serve as theragnostic biomarkers to monitor disease progression and therapeutic response. Thus, TP-41 has potential not only as a therapeutic agent but also within a theragnostic framework, where patient stratification and treatment efficacy could be guided by MGO-related biomarker profiling.

In summary, through DL-guided drug discovery, we identified a novel compound, TP-41, that targets MGO activity for AD treatment. We also demonstrated the feasibility of DL-guided drug discovery in a unique human disease, where a DL-based drug screening strategy had not previously been established owing to a lack of biochemical assay screening data for training DL models. Finally, we propose that TP-41 is a suitable lead compound for further research and development of symptom-ameliorating AD medications.

## Supplementary Material

Supplementary figures and tables.

## Abbreviations

AD: Alzheimer's disease
AGEs: advanced glycation end products
APP: amyloid precursor protein
AUROC: area under the receiver operating characteristic curve
BM: Barnes maze test
DL: deep learning
ELISA: enzyme-linked immunosorbent assay
EPM: elevated plus maze test
FST: forced swim test
GR: glucocorticoid receptor
HPA: hypothalamic-pituitary-adrenal
ML: machine learning
mAβ_1-42_: monomeric amyloid-β_1-42_
NORT: novel object recognition test
oAβ: oligomeric amyloid-β
OFT: open field test
PCC: Pearson's correlation coefficient
p-mTau: phosphorylated monomeric tau
p-oTau: phosphorylated oligomeric tau
RAGE: receptor for advanced glycation end products
RF: random forest
RMSE: root mean square error
ROS: reactive oxygen species
SVR: support vector regression
TP-41: spermidine-conjugated indole derivative compound
Trp: tryptophan
TST: tail suspension test
5xFAD: five familial Alzheimer's disease mutations model
