# Supplementary material for: Deep learning identifies TP-41 for methylglyoxal scavenging in Alzheimer's treatment
Source: Theranostics. 2026 Jan 1;16(3):1103–22. doi: 10.7150/thno.111550 (PMC12679084; doi:10.7150/thno.111550)
Supplement: Supplementary file 1 — Supplementary figures and tables. [file thnov16p1103s1.pdf]

Deep learning identifies TP-41 for methylglyoxal scavenging in Alzheimer's treatment

Park and Hong *et al*.

Supplementary Information

Tables S1 through S9

Figures S1 through S8

Raw image files of Western blotting assay data in Figure 4J, Figure 5F, and Figure 6F.

Supplementary References

Table S1. Model architecture of DeepMGO for MGO affinity prediction.

Layer (type)	Output Shape	Param #	Connected to
**drug_input (InputLayer)**	(None, 2756, 1)	0	
**conv1d_1 (Conv1D)**	(None, 853, 50)	10050	drug_input[0][0]
**max_pooling1d_1 (MaxPooling1D)**	(None, 171, 50)	0	conv1d_1[0][0]
**conv1d_2 (Conv1D)**	(None, 35, 30)	75030	max_pooling1d_1[0][0]
**max_pooling1d_2 (MaxPooling1D)**	(None, 4, 30)	0	conv1d_2[0][0]
**flatten_1 (Flatten)**	(None, 120)	0	max_pooling1d_2[0][0]
**dense_1 (Dense)**	(None, 100)	12100	flatten_1[0][0]
**dropout_1 (Dropout)**	(None, 100)	0	dense_1[0][0]
**dense_2 (Dense)**	(None, 300)	30300	dropout_1[0][0]
**dropout_2 (Dropout)**	(None, 300)	0	dense_2[0][0]
**reshape_1 (Reshape)**	(None, 300, 1)	0	dropout_2[0][0]
**conv1d_3 (Conv1D)**	(None, 151, 30)	4530	reshape_1[0][0]
**max_pooling1d_3 (MaxPooling1D)**	(None, 75, 30)	0	conv1d_3[0][0]
**dropout_3 (Dropout)**	(None, 75, 30)	0	max_pooling1d_3[0][0]
**conc_input (InputLayer)**	(None, 1, 1)	0	
**flatten_2 (Flatten)**	(None, 2250)	0	dropout_3[0][0]
**flatten_3 (Flatten)**	(None, 1)	0	conc_input[0][0]
**dropout_4 (Dropout)**	(None, 2250)	0	flatten_2[0][0]
**dropout_5 (Dropout)**	(None, 1)	0	flatten_3[0][0]
**concatenate_1 (Concatenate)**	(None, 2251)	0	dropout_4[0][0]
			dropout_5[0][0]
**dense_3 (Dense)**	(None, 40)	90080	concatenate_1[0][0]
**Prediction (Dense)**	(None, 1)	41	dense_3[0][0]
**Total params: 222,131**			
**Trainable params: 222,131**			
**Non-trainable params: 0**			

Table S2. Model architecture of DeepIC50 for MGO affinity prediction.

Layer (type)	Output Shape	Param #	Connected to
**inputs (InputLayer)**	(None, 2756, 1)	0	
**conv1d_1 (Conv1D)**	(None, 2756, 16)	192	inputs[0][0]
**batch_normalization_1 (Batch_normalization)**	(None, 2756, 16)	64	conv1d_1[0][0]
**activation_1 (Activation)**	(None, 2756, 16)	0	batch_normalization_1[0][0]
**conv1d_2 (Conv1D)**	(None, 2756, 16)	2832	activation_1[0][0]
**batch_normalization_2 (Batch_normalization)**	(None, 2756, 16)	64	conv1d_2[0][0]
**activation_2 (Activation)**	(None, 2756, 16)	0	batch_normalization_2[0][0]
**max_pooling1d_1 (MaxPooling1D)**	(None, 1378, 16)	0	activation_2[0][0]
**conv1d_3 (Conv1D)**	(None, 1378, 32)	5664	max_pooling1d_1[0][0]
**batch_normalization_3 (Batch_normalization)**	(None, 1378, 32)	128	conv1d_3[0][0]
**activation_3 (Activation)**	(None, 1378, 32)	0	batch_normalization_3[0][0]
**conv1d_4 (Conv1D)**	(None, 1378, 32)	11296	activation_3[0][0]
**max_pooling1d_2 (MaxPooling1D)**	(None, 689, 32)	0	conv1d_4[0][0]
**batch_normalization_4 (Batch_normalization)**	(None, 689, 32)	128	max_pooling1d_2[0][0]
**activation_4 (Activation)**	(None, 689, 32)	0	batch_normalization_4[0][0]
**conv1d_5 (Conv1D)**	(None, 689, 64)	22592	activation_4[0][0]
**batch_normalization_5 (Batch_normalization)**	(None, 689, 64)	256	conv1d_5[0][0]
**activation_5 (Activation)**	(None, 689, 64)	0	batch_normalization_5[0][0]
**max_pooling1d_3 (MaxPooling1D)**	(None, 345, 64)	0	activation_5[0][0]
**conv1d_6 (Conv1D)**	(None, 345, 64)	45120	max_pooling1d_3[0][0]
**batch_normalization_6 (Batch_normalization)**	(None, 345, 64)	256	conv1d_6[0][0]
**activation_6 (Activation)**	(None, 345, 64)	0	batch_normalization_6[0][0]
**max_pooling1d_4 (MaxPooling1D)**	(None, 173, 64)	0	activation_6[0][0]
**flatten_1 (Flatten)**	(None, 11072)	0	max_pooling1d_4[0][0]
**dense_1 (Dense)**	(None, 1024)	11338752	flatten_1[0][0]
**batch_normalization_7 (Batch_normalization)**	(None, 1024)	4096	dense_1[0][0]
**activation_7 (Activation)**	(None, 1024)	0	batch_normalization_7[0][0]
**dropout_1 (Dropout)**	(None, 1024)	0	activation_7[0][0]
**dense_2 (Dense)**	(None, 2048)	2099200	dropout_1[0][0]
**batch_normalization_8 (Batch_normalization)**	(None, 2048)	8192	dense_2[0][0]
**activation_8 (Activation)**	(None, 2048)	0	batch_normalization_8[0][0]
**dropout_2 (Dropout)**	(None, 2048)	0	activation_8[0][0]
**dense_3 (Dense)**	(None, 4096)	8392704	dropout_2[0][0]

**batch_normalization_9 (Batch_normalization)**	(None, 4096)	16384	dense_3[0][0]
**activation_9 (Activation)**	(None, 4096)	0	batch_normalization_9[0][0]
**dropout_3 (Dropout)**	(None, 4096)	0	activation_9[0][0]
**dense_4 (Dense)**	(None, 2048)	8390656	dropout_3[0][0]
**batch_normalization_10 (Batch_normalization)**	(None, 2048)	8192	dense_4[0][0]
**activation_10 (Activation)**	(None, 2048)	0	batch_normalization_10[0][0]
**dropout_4 (Dropout)**	(None, 2048)	0	activation_10[0][0]
**dense_5 (Dense)**	(None, 1024)	2098176	dropout_4[0][0]
**batch_normalization_11 (Batch_normalization)**	(None, 1024)	4096	dense_5[0][0]
**activation_11 (Activation)**	(None, 1024)	0	batch_normalization_11[0][0]
**inputs_drug (InputLayer)**	(None, 1, 1)	0	
**dropout_5 (Dropout)**	(None, 1024)	0	inputs__drug[0][0]
**flatten_2 (Flatten)**	(None, 1)	0	dropout_5[0][0]
**concatenate_1 (Concatenate)**	(None, 1025)	0	flatten_2[0][0]
**predictions (Dense)**	(None, 1)	1026	concatenate_1[0][0]
**Total params: 32,450,066**			
**Trainable params: 32,429,138**			
**Non-trainable params: 20,928**			

Table S3. Model architecture of ResNet18 for MGO affinity prediction.

Layer (type)	Output Shape	Param #	Connected to
**inputs (InputLayer)**	(None, 2756, 1)	0	
**conv1d_21 (Conv1D)**	(None, 1378, 64)	512	inputs[0][0]
**batch_normalization_21 (Batch_normalization)**	(None, 1378, 64)	256	conv1d_21[0][0]
**activation_18 (Activation)**	(None, 1378, 64)	0	batch_normalization_21[0][0]
**max_pooling1d_2 (MaxPooling1D)**	(None, 689, 64)	0	activation_18[0][0]
**conv1d_22 (Conv1D)**	(None, 689, 64)	12352	max_pooling1d_2[0][0]
**batch_normalization_22 (Batch_normalization)**	(None, 689, 64)	256	conv1d_22[0][0]
**activation_19 (Activation)**	(None, 689, 64)	0	batch_normalization_22[0][0]
**conv1d_23 (Conv1D)**	(None, 689, 64)	12352	activation_19[0][0]
**batch_normalization_23 (Batch_normalization)**	(None, 689, 64)	256	conv1d_23[0][0]
**add_9 (Add)**	(None, 689, 64)	0	batch_normalization_23[0][0]
			max_pooling1d_2[0][0]
**activation_20 (Activation)**	(None, 689, 64)	0	add_9[0][0]
**conv1d_24 (Conv1D)**	(None, 689, 64)	12352	activation_20[0][0]
**batch_normalization_24 (Batch_normalization)**	(None, 689, 64)	256	conv1d_24[0][0]
**activation_21 (Activation)**	(None, 689, 64)	0	batch_normalization_24[0][0]
**conv1d_25 (Conv1D)**	(None, 689, 64)	12352	activation_21[0][0]
**batch_normalization_25 (Batch_normalization)**	(None, 689, 64)	256	conv1d_25[0][0]
**add_10 (Add)**	(None, 689, 64)	0	batch_normalization_25[0][0]
			activation_20[0][0]
**activation_22 (Activation)**	(None, 689, 64)	0	add_10[0][0]
**conv1d_27 (Conv1D)**	(None, 345, 128)	24704	activation_22[0][0]
**batch_normalization_27 (Batch_normalization)**	(None, 345, 128)	512	conv1d_27[0][0]
**activation_23 (Activation)**	(None, 345, 128)	0	batch_normalization_27[0][0]
**conv1d_28 (Conv1D)**	(None, 345, 128)	49280	activation_23[0][0]
**conv1d_26 (Conv1D)**	(None, 345, 128)	8320	activation_22[0][0]
**batch_normalization_28 (Batch_normalization)**	(None, 345, 128)	512	conv1d_28[0][0]
**batch_normalization_26 (Batch_normalization)**	(None, 345, 128)	512	conv1d_26[0][0]
**add_11 (Add)**	(None, 345, 128)	0	batch_normalization_28[0][0]
			batch_normalization_26[0][0]
**activation_24 (Activation)**	(None, 345, 128)	0	add_11[0][0]
**conv1d_29 (Conv1D)**	(None, 345, 128)	49280	activation_24[0][0]
**batch_normalization_29 (Batch_normalization)**	(None, 345, 128)	512	conv1d_29[0][0]
**activation_25 (Activation)**	(None, 345, 128)	0	batch_normalization_29[0][0]
**conv1d_30 (Conv1D)**	(None, 345, 128)	49280	activation_25[0][0]
**batch_normalization_30 (Batch_normalization)**	(None, 345, 128)	512	conv1d_30[0][0]
**add_12 (Add)**	(None, 345, 128)	0	batch_normalization_30[0][0]
			activation_24[0][0]
**activation_26 (Activation)**	(None, 345, 128)	0	add_12[0][0]
**conv1d_32 (Conv1D)**	(None, 173, 256)	98560	activation_26[0][0]
**batch_normalization_32 (Batch_normalization)**	(None, 173, 256)	1024	conv1d_32[0][0]
**activation_27 (Activation)**	(None, 173, 256)	0	batch_normalization_32[0][0]

**conv1d_33 (Conv1D)**	(None, 173, 256)	196864	activation_27[0][0]
**conv1d_31 (Conv1D)**	(None, 173, 256)	33024	activation_26[0][0]
**batch_normalization_33 (Batch_normalization)**	(None, 173, 256)	1024	conv1d_33[0][0]
**batch_normalization_31 (Batch_normalization)**	(None, 173, 256)	1024	conv1d_31[0][0]
**add_13 (Add)**	(None, 173, 256)	0	batch_normalization_33[0][0]
			batch_normalization_31[0][0]
**activation_28 (Activation)**	(None, 173, 256)	0	add_13[0][0]
**conv1d_34 (Conv1D)**	(None, 173, 256)	196864	activation_28[0][0]
**batch_normalization_34 (Batch_normalization)**	(None, 173, 256)	1024	conv1d_34[0][0]
**activation_29 (Activation)**	(None, 173, 256)	0	batch_normalization_34[0][0]
**conv1d_35 (Conv1D)**	(None, 173, 256)	196864	activation_29[0][0]
**batch_normalization_35 (Batch_normalization)**	(None, 173, 256)	1024	conv1d_35[0][0]
**add_14 (Add)**	(None, 173, 256)	0	batch_normalization_35[0][0]
			activation_28[0][0]
**activation_30 (Activation)**	(None, 173, 256)	0	add_14[0][0]
**conv1d_37 (Conv1D)**	(None, 87, 512)	393728	activation_30[0][0]
**batch_normalization_37 (Batch_normalization)**	(None, 87, 512)	2048	conv1d_37[0][0]
**activation_31 (Activation)**	(None, 87, 512)	0	batch_normalization_37[0][0]
**conv1d_38 (Conv1D)**	(None, 87, 512)	786944	activation_31[0][0]
**conv1d_36 (Conv1D)**	(None, 87, 512)	131584	activation_30[0][0]
**batch_normalization_38 (Batch_normalization)**	(None, 87, 512)	2048	conv1d_38[0][0]
**batch_normalization_36 (Batch_normalization)**	(None, 87, 512)	2048	conv1d_36[0][0]
**add_15 (Add)**	(None, 87, 512)	0	batch_normalization_38[0][0]
			batch_normalization_36[0][0]
**activation_32 (Activation)**	(None, 87, 512)	0	add_15[0][0]
**conv1d_39 (Conv1D)**	(None, 87, 512)	786944	activation_32[0][0]
**batch_normalization_39 (Batch_normalization)**	(None, 87, 512)	2048	conv1d_39[0][0]
**activation_33 (Activation)**	(None, 87, 512)	0	batch_normalization_39[0][0]
**conv1d_40 (Conv1D)**	(None, 87, 512)	786944	activation_33[0][0]
**batch_normalization_40 (Batch_normalization)**	(None, 87, 512)	2048	conv1d_40[0][0]
**add_16 (Add)**	(None, 87, 512)	0	batch_normalization_40[0][0]
			activation_32[0][0]
**activation_34 (Activation)**	(None, 87, 512)	0	add_16[0][0]
**average_pooling1d_2 (AveragePooling)**	(None, 43, 512)	0	activation_34[0][0]
**inputs__drug (InputLayer)**	(None, 1, 1)	0	
**flatten_3 (Flatten)**	(None, 22016)	0	average_pooling1d_2[0][0]
**flatten_4 (Flatten)**	(None, 1)	0	inputs__drug[0][0]
**concatenate_2 (Concatenate)**	(None, 22017)	0	flatten_3[0][0]
			flatten_4[0][0]
**predictions (Dense)**	(None, 1)	22018	concatenate_2[0][0]
**Total params: 3,880,322**			
**Trainable params: 3,870,722**			
**Non-trainable params: 9,600**			

Table S4. ML models and their parameters per selected features (%).

Methods (parameters)	Selected features (%)					
	**10**	**30**	**50**	**70**	**90**	**100**
**Lasso (Alpha)**	0.001					
**Ridge (Alpha)**	0.001	0.01				0.001
**SVR (C / Gamma)**	1 / 0.001					
**RF (number of trees)**	10					

Table S5. The compounds, identified by their PubChem IDs in the independent data, had their MGO or AGE binding states (active or inactive) confirmed through literatures.

Compounds	PubChem ID	State (active/inactive)	Reference
Vitexin	5280441	active	[1]
isovitexin	162350	active	
palmatine	19009	active	[2]
glimerpiride	3476	active	
metformin	4091	active	
2,3,5,4'-tetrahydroxystilbene 2-O-β-D-glucoside (THSG)	73981748	active	[3]
pyridoxamine (Pyridorin)	1052	active	
kaempferol	5280863	active	[4]
(+)-gallocatechin	65084	active	[5]
(-)-gallocatechin	9882981	active	
(-)-epigallocatechin	72277	active	
(+)-catechin	9064	active	
(-)-catechin	73160	active	
epicatechin-(4β-8)-gallocatechin	14284599	active	
epigallocatechin-(4β-8)-epicatechin	442678	active	
procyanidin B2	122738	active	
(-)-epicatechin	72276	active	
chebulic acid	71308174	active	[6]
quercetin	5280343	active	[7]
sulfuretin	5281295	active	[8]
Butein	5281222	active	
protocatechuic acid	72	inactive	
ethyl gallate	13250	inactive	
Fustin	5317435	inactive	
Morin	16219651	inactive	
Fisetin	5281614	inactive	
pentagalloyl glucose	65238	inactive	
5,6-dehydrokawain (DK)	5273621	active	[9]
dihydro-5,6-dehydrokawain (DDK)	160673	active	
8(17),12-labdadiene-15,16-dial (labdadiene)	9904510	active	
aucubin	91458	active	[10]
isoferulic acid (IFA)	736186	active	[11]
ferulic acid (FA)	445858	active	[12]
curcumin	969516	active	[13]
chlorogenic acid	1794427	active	[14]
3,5-di-O-caffeoylquinic acid	6474310	active	
resveratrol	445154	active	[15]
genistein	5280961	active	[16]
corosolic acid	6918774	active	[17]

ellagic acid	5281855	active	
ursolic acid	64945	active	
epigallocatechin gallate	65064	active	[18]
epicatechin gallate	107905	active	
eriodictyol	440735	active	[19]
naringenin	932	active	
aloin	9866696	active	
anthraquinone	6780	active	
chrysophanol	10208	active	
emodin	3220	active	
physcion	10639	active	
rhein	10168	active	
phloretin	4788	active	[20]
phloridzin	6072	active	
cyanidin-3-rutinoside (C3R)	14034151	active	[21]
caffeic acid	689043	active	[22]
berberine	2353	active	[23]
genistin	5281377	inactive	[24]
(-)-medicarpin	336327	inactive	
(-)-glycinol	129648	inactive	
(-)-tuberosin	14630495	inactive	
3-hydroxy-5-methoxybiphenyl	12000323	inactive	[25]

Table S6. R^2 values in DL and ML models using each selected features (%) in the test set.

Methods	Selected features (%)					
	100	90	70	50	30	10
**DeepMGO**	0.939	**0.952***	0.943	0.913	0.79	0.893
**ResNet18**	-2.754	0.156	0.003	-0.415	0.669	0.821
**DeepIC50**	0.640	0.753	0.615	0.794	0.842	0.779
**Lasso**	-0.026	-0.026	-0.026	-0.091	-0.029	-0.073
**Ridge**	-3.530	0.667	-4.403	0.511	0.082	-0.256
**SVR**	0.158	0.158	0.158	0.146	0.116	0.082
**Random forest**	0.889	0.797	0.885	0.864	0.906	0.949

*Note that models with an R^2 of 0.95 or higher are indicated in bold.

Table S7. Log_2(RMSE) values in DL and ML models using each selected features (%) in the test set.

Methods	Selected features (%)					
	**100**	**90**	**70**	**50**	**30**	**10**
**DeepMGO**	-3.921	**-4.083**	-3.966	-3.662	-3.023	-3.506
**ResNet18**	-0.943	-2.017	-1.900	-1.648	-2.690	-3.133
**DeepIC50**	-2.635	-2.911	-2.582	-3.035	-3.224	-2.989
**Lasso**	-1.878	-1.878	-1.878	-1.837	-1.878	-1.847
**Ridge**	-0.808	-2.690	-0.680	-2.411	-1.960	-1.732
**SVR**	-2.023	-2.023	-2.023	-2.012	-1.989	-1.960
**Random forest**	-3.474	-3.047	-3.458	-3.336	-3.608	**-4.059**

*Note that models with a Log_2(RMSE) of -4.000 or lower are indicated in bold.

Table S8. Predicted MGO affinity scores of the compounds in application data using DeepMGO.

Compounds	Concentration (μM)	Predicted MGO affinity score
TP-41	500	2.692
TP-15	1000	2.668
TP-3	1000	2.665
TP-25 (5-HT)	1000	2.656
TP-20	1000	2.395
TP-11	1000	2.376
TP-18	400	2.344
TP-39	500	2.245
TP-26	1000	2.021
TP-16	1000	2.000
TP-8	1000	1.990
TP-20	400	1.966
TP-3	400	1.930
TP-41	100	1.928
TP-22	400	1.799
TP-22	1000	1.798
TP-14	1000	1.752
TP-40	500	1.711
TP-21	1000	1.677
TP-25 (5-HT)	400	1.669
TP-8	400	1.613
TP-21	400	1.562
TP-17	1000	1.550
TP-36	1000	1.543
TP-23 (Trp)	1000	1.536
TP-12	1000	1.524
TP-10	1000	1.446
TP-27	1000	1.356
TP-3	100	1.272
TP-20	100	1.260
TP-36	500	1.214
TP-22	100	1.196
TP-34	1000	1.177
TP-31	1000	1.169
TP-25 (5-HT)	100	1.161
TP-32	1000	1.160
TP-33	1000	1.085
TP-23 (Trp)	400	1.023

TP-13	1000	0.999
TP-21	100	0.931
TP-44	100	0.908
TP-28	1000	0.882
TP-1(5-HTP)	400	0.763
TP-29	1000	0.754
TP-24 (Tryptamine)	1000	0.734
TP-1(5-HTP)	1000	0.664
TP-24 (Tryptamine)	400	0.646
TP-42	100	0.546
TP-35	1000	0.517
TP-38	500	0.356
TP-36	100	0.341
TP-30	1000	0.314
TP-23 (Trp)	100	0.302
TP-1(5-HTP)	100	0.245
TP-9	1000	0.229
TP-2	100	0.155
TP-5	100	0.123
TP-2	400	0.105
TP-37	400	0.105
TP-7	100	0.074
TP-6	400	0.074
TP-19	100	0.041
TP-24 (Tryptamine)	100	0.038
TP-37	100	0.035
TP-7	400	0.024
TP-43	100	0.012
TP-4	100	0.008
TP-2	1000	0.006
TP-37	1000	0.005
TP-5	100	0.003

Table S9. Description and physicochemical interpretation of key PaDEL molecular descriptors. These features were identified as significant contributors to model predictions in the SHAP analysis for Figure S7 and represent various molecular properties, including size, complexity, polarity, 3D shape, and electronic characteristics.

Feature	Description	Role and Meaning
**nT6HeteroRing**	Number of 6-membered heteroaromatic rings	Number of 6-membered rings (includes counts from fused rings) containing heteroatoms (N, O, P, S, or halogens)
**VP-1**	Valence path, order 1	A topological descriptor that evaluates the connectivity and branching of a molecule's carbon skeleton. It is based on the valence state of atoms and the paths between them.
**BCUTp-1h**	Nlow highest polarizability weighted BCUTS	A 3D descriptor that combines atomic properties (specifically, polarizability) with the molecule's connectivity to represent its overall shape and electronic properties.
**E-state Descriptors (max... or min…)**	Atom-type E-state values	A composite value that represents the electronic properties and local environment of a specific atom or functional group (e.g., the -OH group in minHssOH). These are key for predicting intermolecular interactions like hydrogen bonds.
**ETA Descriptors**	Extended Topochemical Atom descriptors	A complex descriptor combining the molecule's topology (atomic connectivity) and electronic properties to describe the chemical properties and reactivity of specific molecular regions.
**ATS Descriptors**	Autocorrelation of Topological Structure (2D)	These descriptors describe how a specific property is distributed across a molecule's topological structure. Using an autocorrelation function like Moreau-Broto Autocorrelation, they correlate physicochemical properties (e.g., atomic weight, electronegativity) between atom pairs at specific topological distances. This provides a numerical summary of the molecule's structural complexity and property distribution, making them highly valuable for modeling molecular properties.
**SpMin/ SpMax**	Burden Modified Eigen values descriptor	SpMin/SpMax are descriptors calculated from the eigenvalues of the Burden matrix. They are derived from a molecule's 3D structure and weighted by various atomic properties such as mass, volume, electronegativity, and polarizability. These descriptors are also classified similarly to 3D-MoRSE descriptors and are used to represent the overall molecular shape, size, and electronic properties.
**PubchemFP...**	PubChem Substructure Fingerprint	A binary value (0 or 1) indicating the presence or absence of a specific chemical substructure or functional group.

Figure S1. Schematic workflow of construction of MGO affinity prediction models. MGO affinity prediction models were constructed with MGO affinity assay data (training and validation data). The performance of MGO affinity prediction models was evaluated using test data. Feature selection was conducted using univariate linear regression.

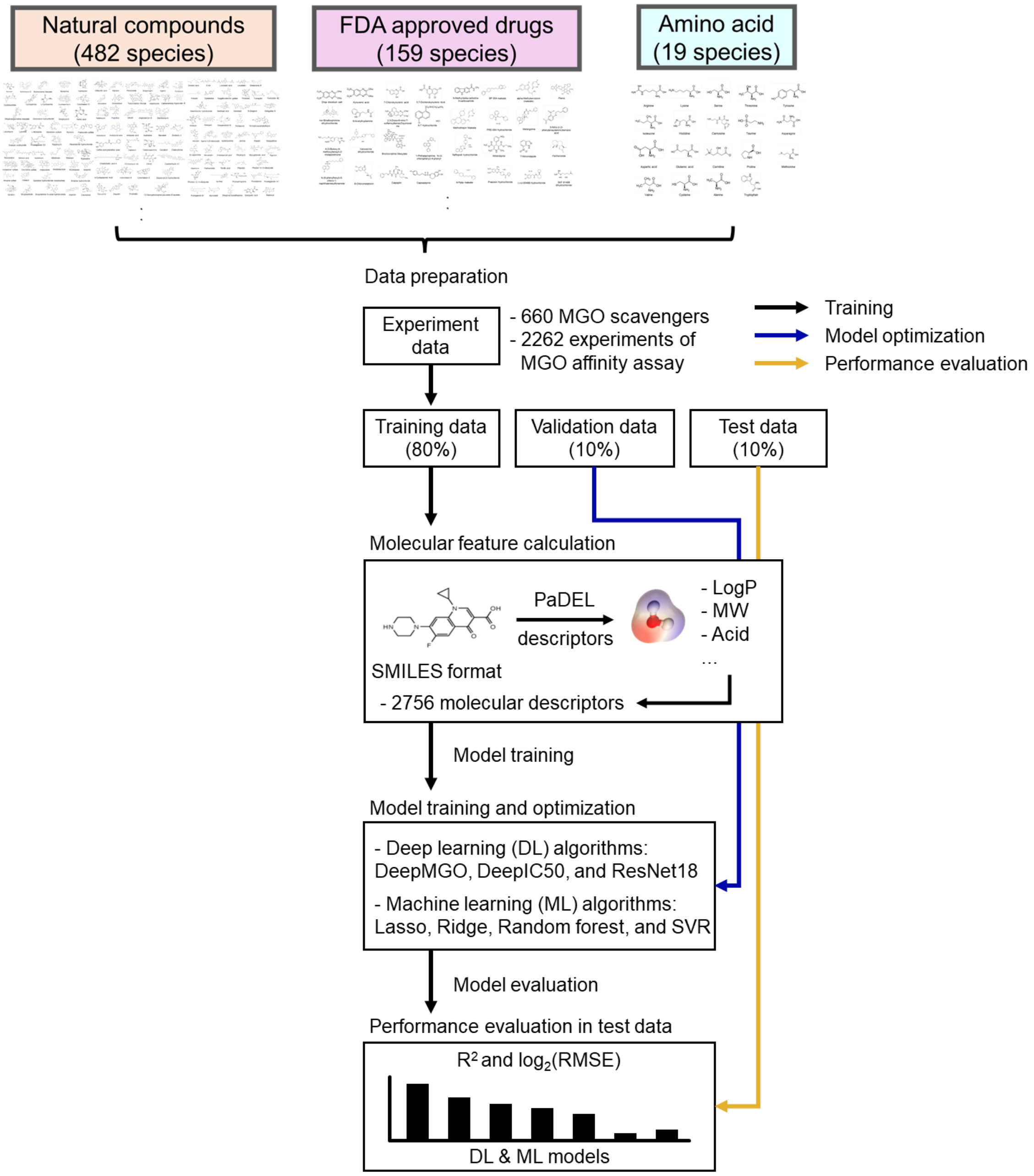

Figure S2. Conceptual architecture of DeepMGO. DeepMGO is based on CNN structure adopted and modified from CDRscan.

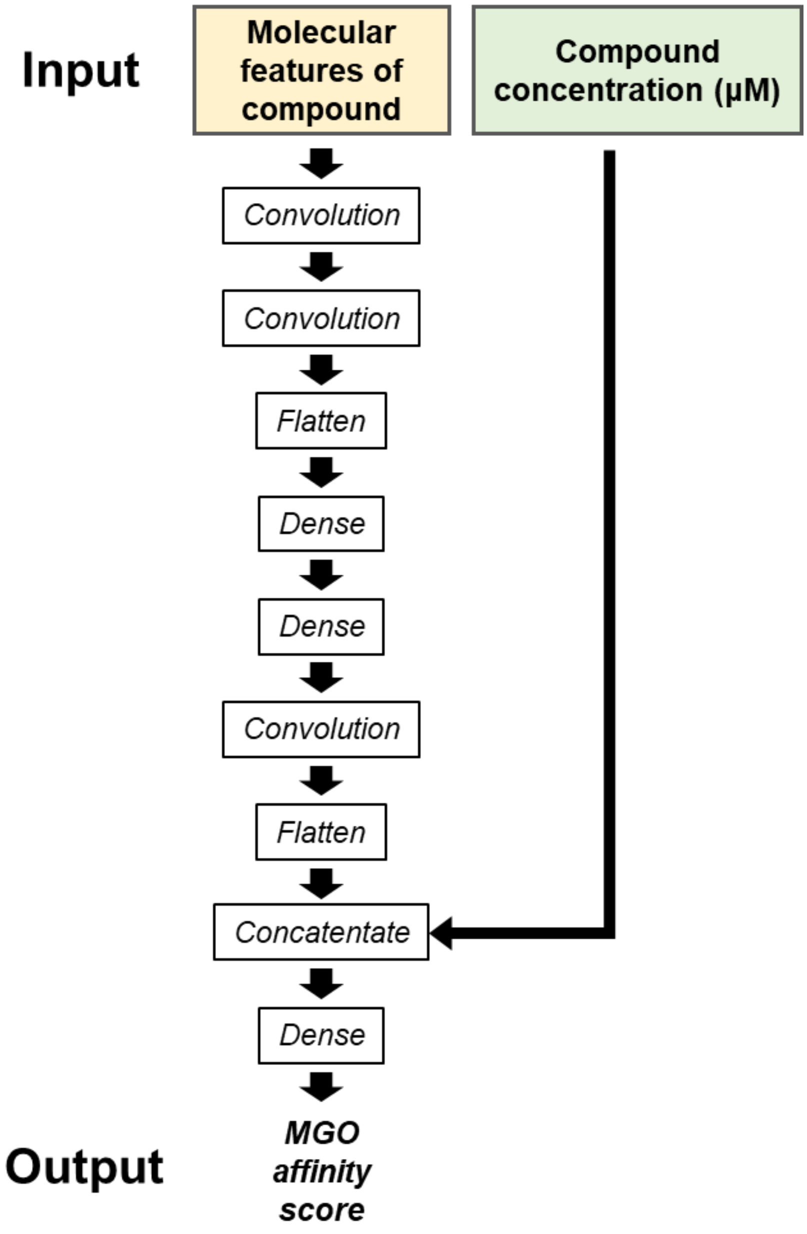

Figure S3. Compounds for identification of putative MGO scavenger to inspect therapeutic effects to AD.

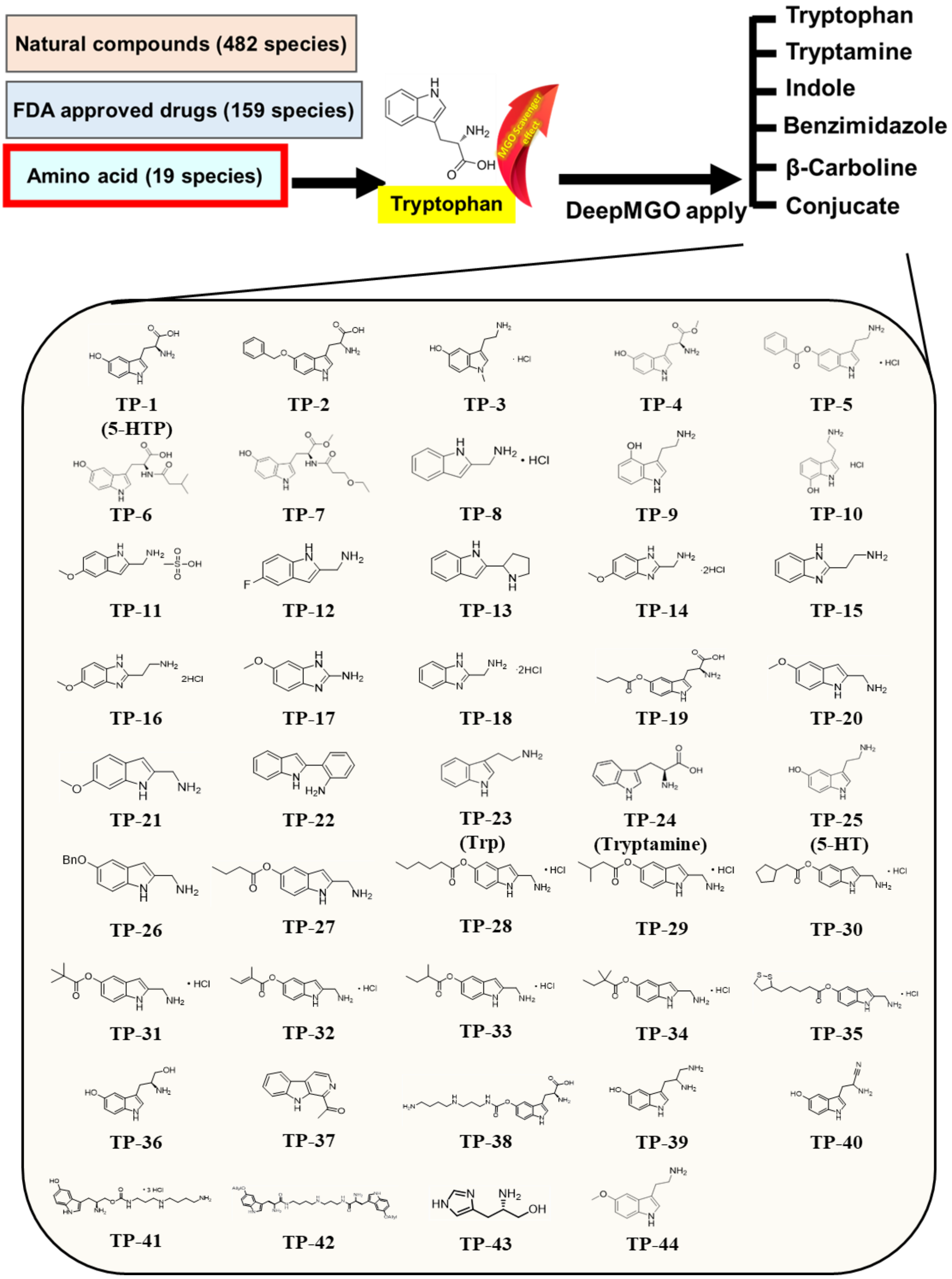

Figure S4. UHPLC–qTOF–MS analysis of TP-41 and Trp in the presence of MGO. Extracted-ion chromatograms (EICs) were obtained under positive ion mode for TP-41 (m/z 378.17), the proposed TP-41–MGO Schiff-base intermediate (m/z 486.21), and a rearranged product (m/z 484.15). For validation, tryptophan (Trp; m/z 205.05) and a putative Trp–MGO adduct (m/z 254.01) were also analyzed. Chromatograms compare compounds alone (TP-41 or Trp), Day 0 (immediately after mixing with MGO), and Day 3 (after incubation). The emergence of intermediate and adduct peaks over time confirms direct chemical quenching of MGO by TP-41 and Trp.

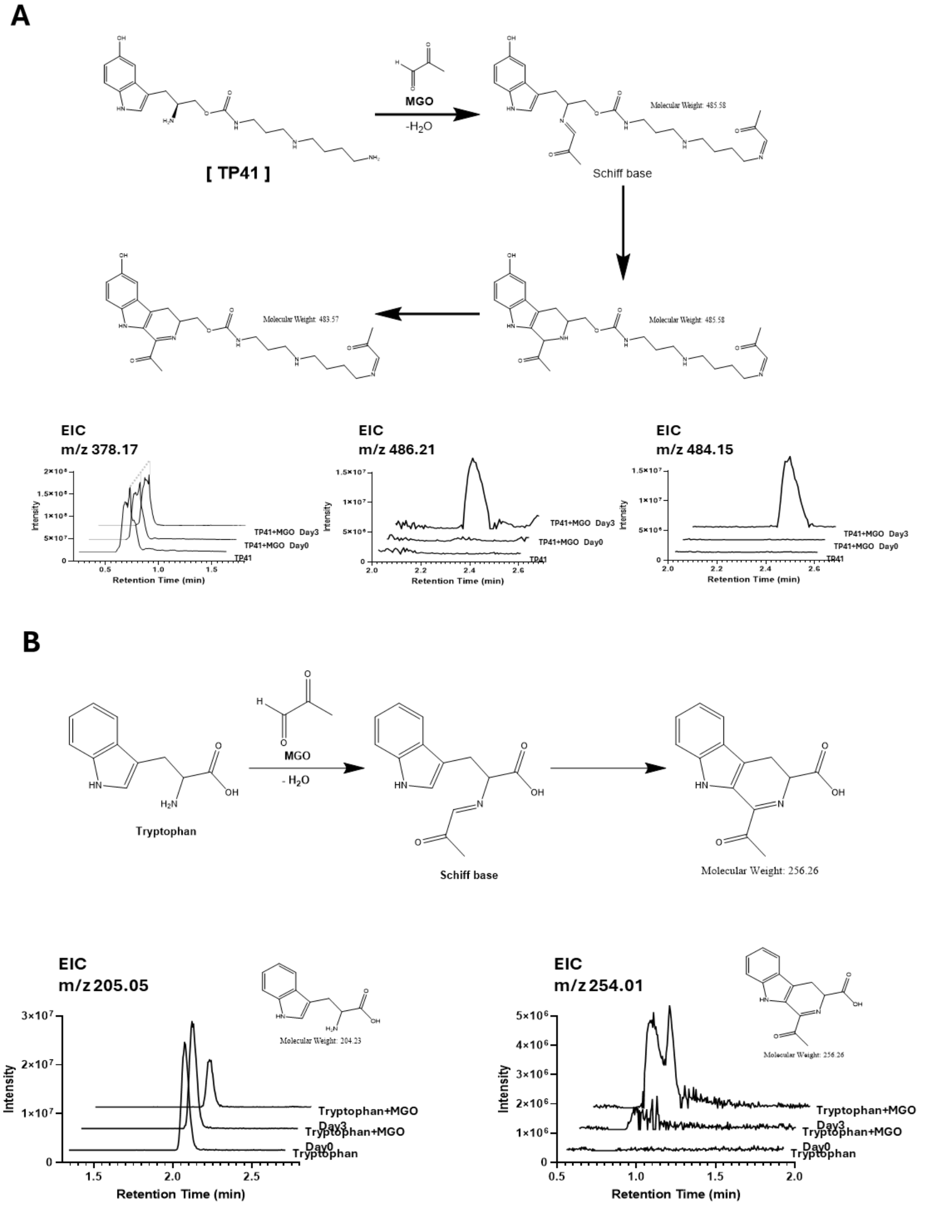

Figure S5. Performance evaluation of MGO affinity prediction models built by using 90% selected features. Negative R^2 value was considered as zero.

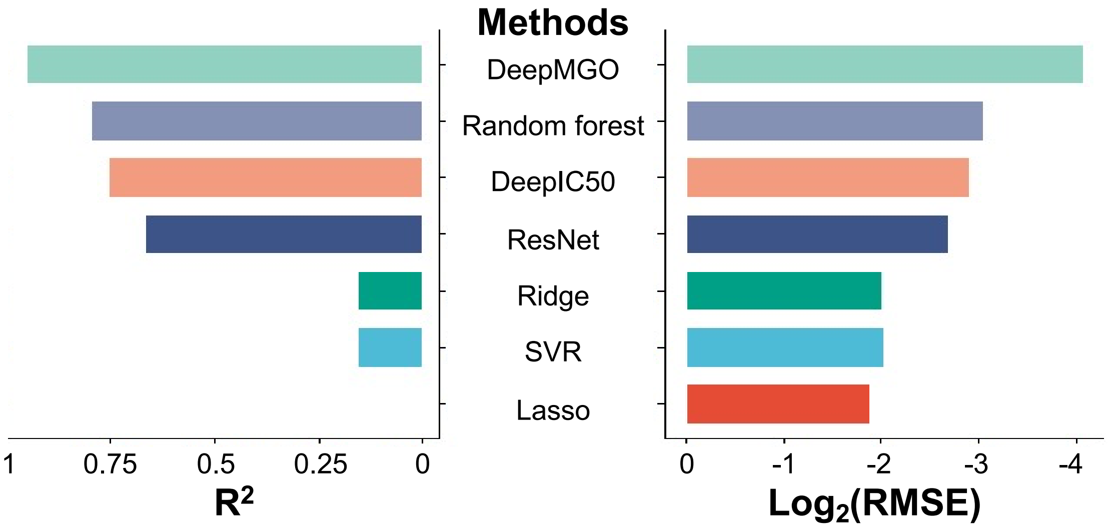

Figure S6. Predicted Blood-Brain Barrier (BBB) Permeability of TP-41 and Reference Compounds. Comparison of the in silico predicted BBB permeability for the lead candidate TP-41 and relevant reference compounds: 5-Hydroxytryptophan (5-HTP, TP-1), and Serotonin (5-HT, TP-25). (A) Predicted LogBB value from the LogBB_Pred model. Higher logBB values indicate higher predicted permeability. The dashed line at LogBB = −1.0 is a cutoff for BBB permeability. (B) Predicted BBB score calculated using the model by ADMET-AI. A score closer to 1 indicates higher predicted permeability.

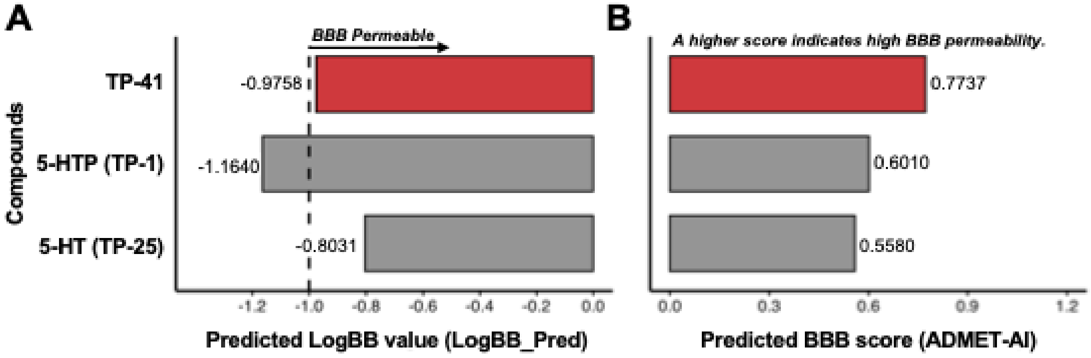

Figure S7. SHAP (SHapley Additive exPlanations) feature importance analysis for DeepMGO, DeepIC50, and ResNet18 on the test set. The bar plots display the top 30 features ranked by their mean absolute SHAP value, which indicates the average impact of each feature on the model's output magnitude. The analysis reveals that DeepMGO utilizes a balanced set of diverse physicochemical features, whereas DeepIC50 heavily relies on E-state descriptors, and ResNet18 exhibits a lack of clearly important features, highlighting its poor interpretability.

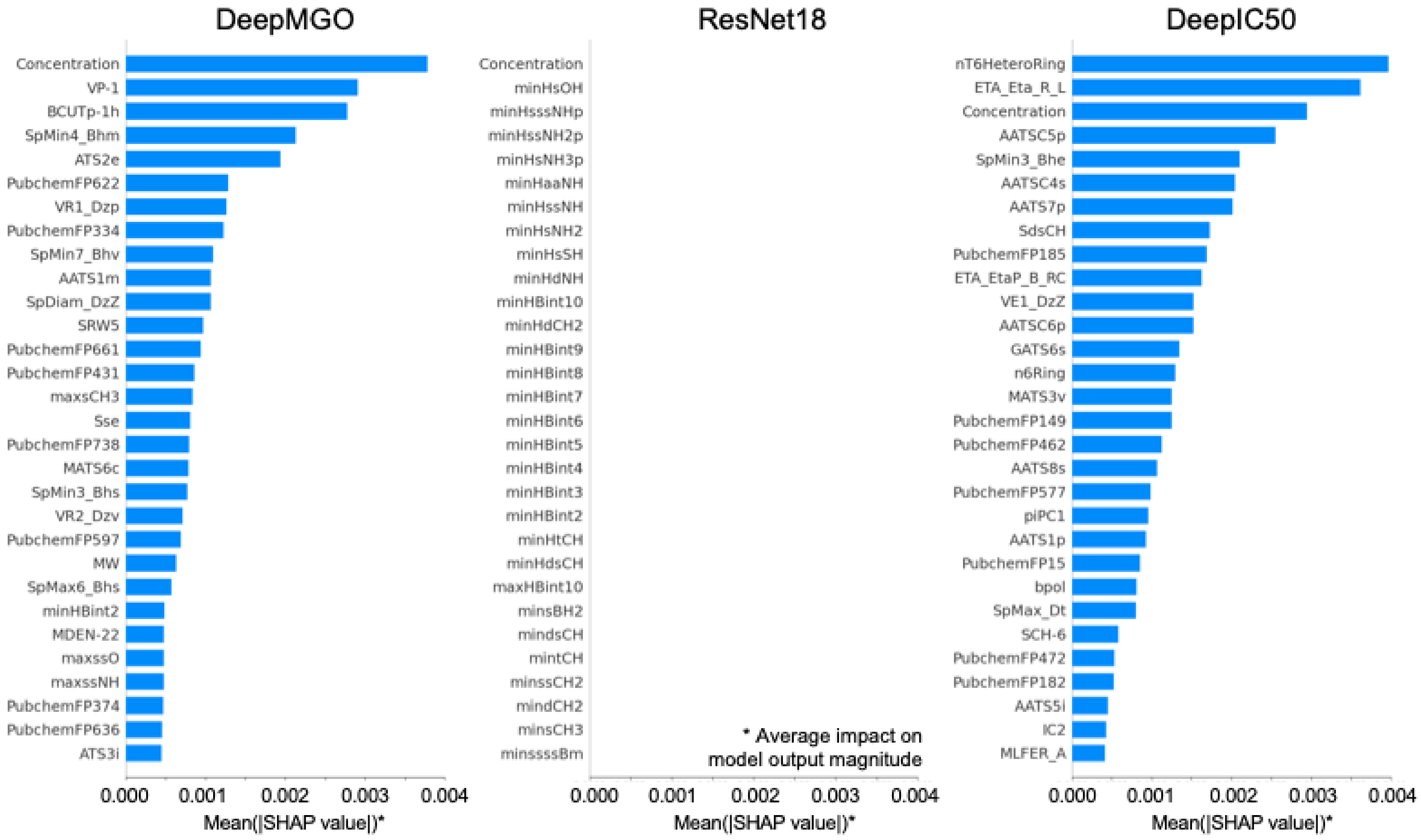

Figure S8. Densitometric analysis of free MGO levels in the MGO-affinity assay, determined by HPLC after derivatization with o-phenylenediamine (o-PD). The chromatographic analysis confirmed that candidate compounds reduced the levels of free MGO, as indicated by decreased formation of the derived compound 2-methylquinoxaline (2-MQ). ###*P* < 0.001 vs. PBS. ****P* < 0.001 vs. MGO group (MGO).

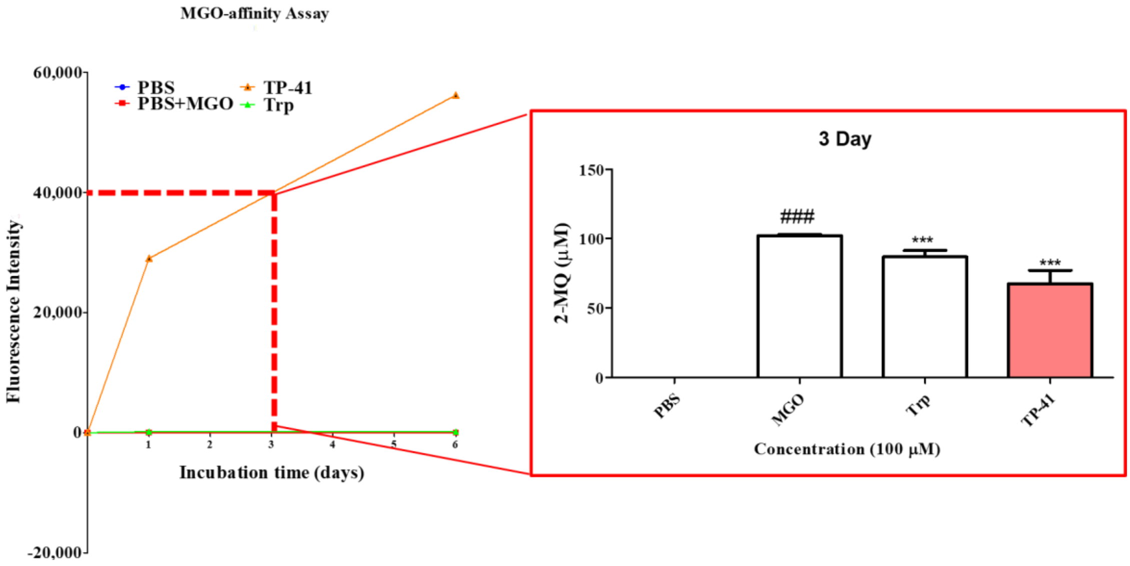

Raw image files of Western blotting assay data in Figure 4J, Figure 5F, and Figure 6F

Figure 4J

RAGE

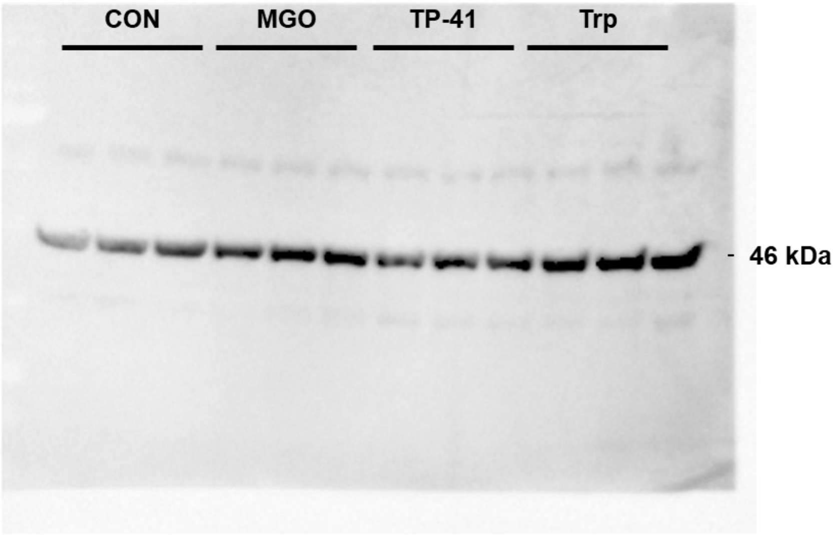

α-Tubulin

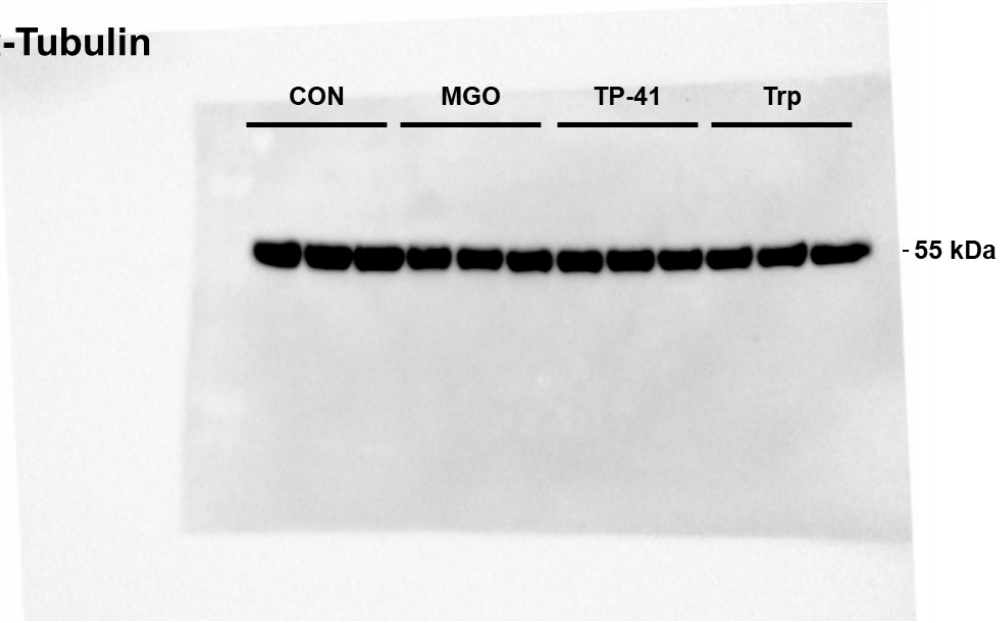

Figure 5F

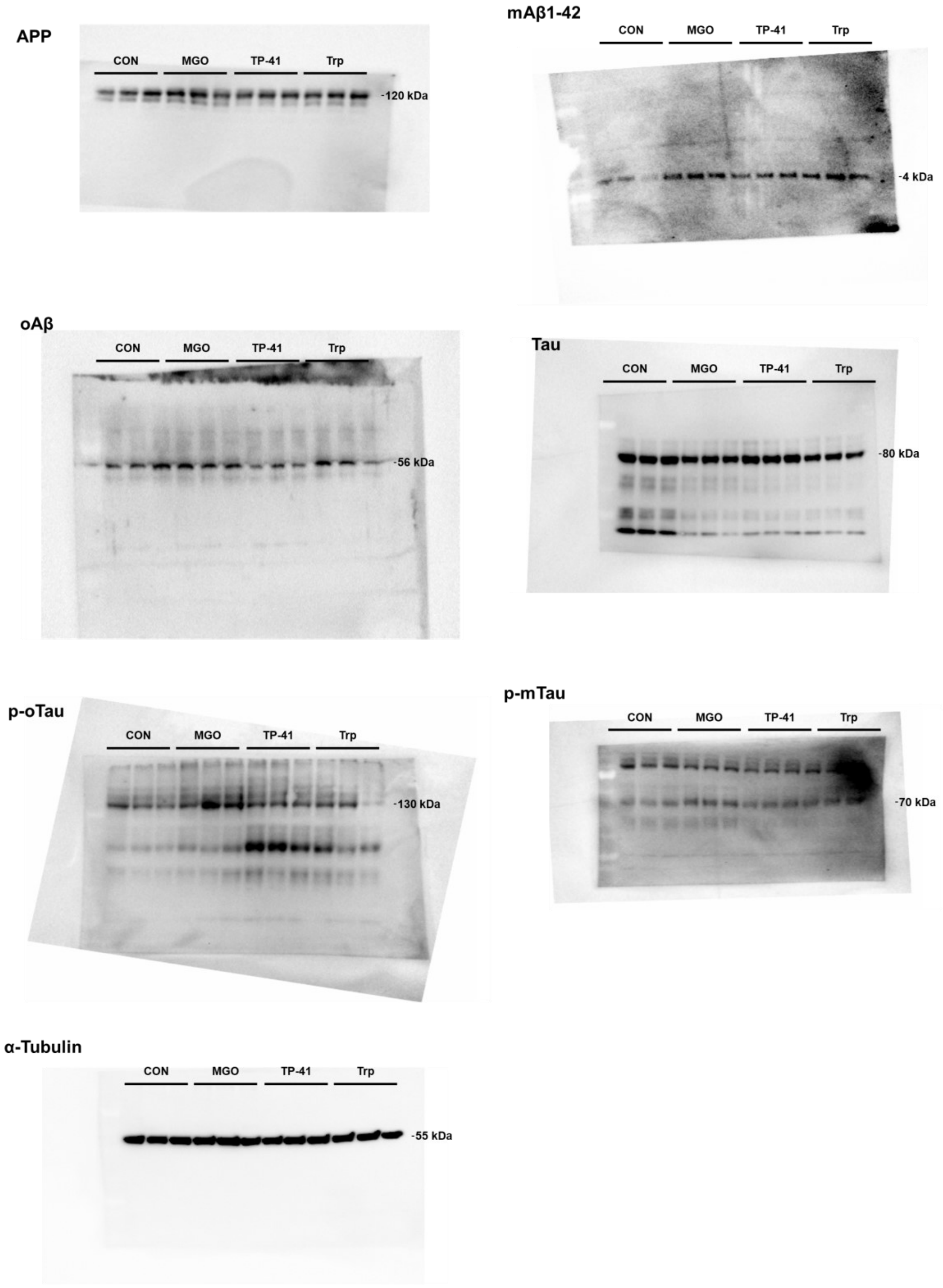

APP
CON MGO TP-41 Trp
-120 kDa
mAβ1-42
CON MGO TP-41 Trp
-4 kDa
oAβ
CON MGO TP-41 Trp
-56 kDa
Tau
CON MGO TP-41 Trp
-80 kDa
p-oTau
CON MGO TP-41 Trp
-130 kDa
p-mTau
CON MGO TP-41 Trp
-70 kDa
α-Tubulin
CON MGO TP-41 Trp
-55 kDa

Figure 6F

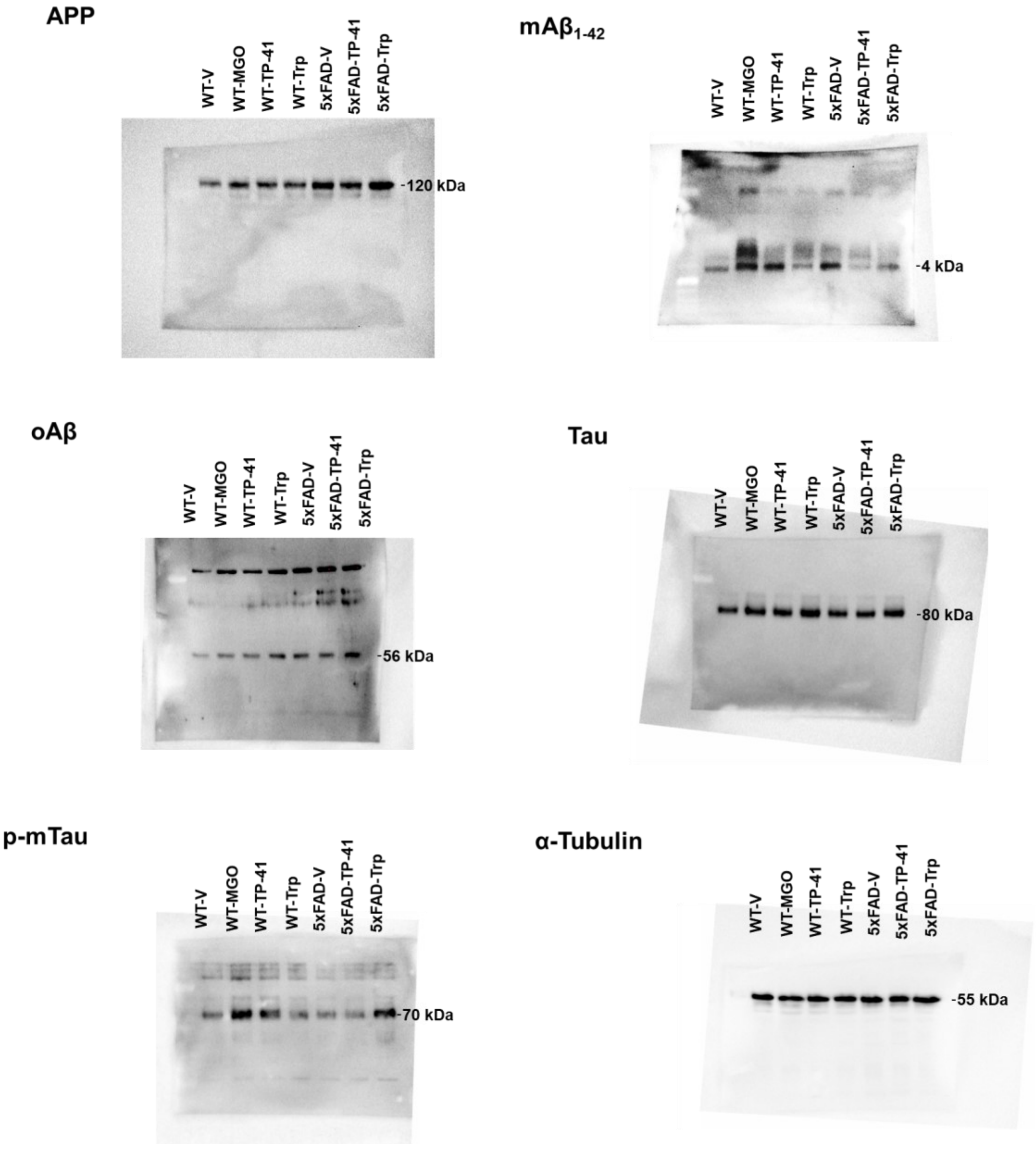
APP
WT-V
WT-MGO
WT-TP-41
WT-Trp
5xFAD-V
5xFAD-TP-41
5xFAD-Trp
-120 kDa
mAβ1-42
WT-V
WT-MGO
WT-TP-41
WT-Trp
5xFAD-V
5xFAD-TP-41
5xFAD-Trp
-4 kDa
oAβ
WT-V
WT-MGO
WT-TP-41
WT-Trp
5xFAD-V
5xFAD-TP-41
5xFAD-Trp
-56 kDa
Tau
WT-V
WT-MGO
WT-TP-41
WT-Trp
5xFAD-V
5xFAD-TP-41
5xFAD-Trp
-80 kDa
p-mTau
WT-V
WT-MGO
WT-TP-41
WT-Trp
5xFAD-V
5xFAD-TP-41
5xFAD-Trp
-70 kDa
α-Tubulin
WT-V
WT-MGO
WT-TP-41
WT-Trp
5xFAD-V
5xFAD-TP-41
5xFAD-Trp
-55 kDa

Supplementary References

1. Peng X, Zheng Z, Cheng K-W, Shan F, Ren G-X, Chen F, et al. Inhibitory effect of mung bean extract and its constituents vitexin and isovitexin on the formation of advanced glycation endproducts. Food Chem. 2008; 106: 475-81.
2. Mridula S, Masroor W, Xavier M, Hui T, Hor Kuan C, Chirara K, et al. Antioxidant and anti-advanced glycation end products formation properties of palmatine. J Pharm Pharmacognosy Res. 2021; 9: 366-78.
3. Lv L, Shao X, Wang L, Huang D, Ho CT, Sang S. Stilbene glucoside from Polygonum multiflorum Thunb.: a novel natural inhibitor of advanced glycation end product formation by trapping of methylglyoxal. J Agric Food Chem. 2010; 58: 2239-45.
4. Yang R, Wang WX, Chen HJ, He ZC, Jia AQ. The inhibition of advanced glycation end-products by five fractions and three main flavonoids from Camellia nitidissima Chi flowers. J Food Drug Anal. 2018; 26: 252-9.
5. Yokozawa T, Nakagawa T. Inhibitory effects of Luobuma tea and its components against glucose-mediated protein damage. Food Chem Toxicol. 2004; 42: 975-81.
6. Lee HS, Koo YC, Suh HJ, Kim KY, Lee KW. Preventive effects of chebulic acid isolated from Terminalia chebula on advanced glycation endproduct-induced endothelial cell dysfunction. J Ethnopharmacol. 2010; 131: 567-74.
7. Li X, Zheng T, Sang S, Lv L. Quercetin inhibits advanced glycation end product formation by trapping methylglyoxal and glyoxal. J Agric Food Chem. 2014; 62: 12152-8.
8. Lee EH, Song DG, Lee JY, Pan CH, Um BH, Jung SH. Inhibitory effect of the compounds isolated from Rhus verniciflua on aldose reductase and advanced glycation endproducts. Biol Pharm Bull. 2008; 31: 1626-30.
9. Chompoo J, Upadhyay A, Kishimoto W, Makise T, Tawata S. Advanced glycation end products inhibitors from Alpinia zerumbet rhizomes. Food Chem. 2011; 129: 709-15.
10. Jung E, Park SB, Jung WK, Kim HR, Kim J. Antiglycation Activity of Aucubin In Vitro and in Exogenous Methylglyoxal Injected Rats. Molecules. 2019; 24: 3653.
11. Arfin S, Siddiqui GA, Naeem A, Moin S. Inhibition of advanced glycation end products by isoferulic acid and its free radical scavenging capacity: An in vitro and molecular docking study. Int J Biol Macromol. 2018; 118: 1479-87.
12. Liu J-l, He Y-l, Wang S, He Y, Wang W-y, Li Q-j, et al. Ferulic acid inhibits advanced glycation end products (AGEs) formation and mitigates the AGEs-induced inflammatory response in HUVEC cells. J Funct Foods. 2018; 48: 19-26.
13. Sun YP, Gu JF, Tan XB, Wang CF, Jia XB, Feng L, et al. Curcumin inhibits advanced glycation end product-induced oxidative stress and inflammatory responses in endothelial cell damage via trapping methylglyoxal. Mol Med Rep. 2016; 13: 1475-86.
14. Kim J, Jo K, Lee IS, Kim CS, Kim JS. The Extract of Aster Koraiensis Prevents Retinal Pericyte Apoptosis in Diabetic Rats and Its Active Compound, Chlorogenic Acid Inhibits AGE Formation and AGE/RAGE Interaction. Nutrients. 2016; 8: 585.

15. Shen Y, Xu Z, Sheng Z. Ability of resveratrol to inhibit advanced glycation end product formation and carbohydrate-hydrolyzing enzyme activity, and to conjugate methylglyoxal. Food Chem. 2017; 216: 153-60.
16. Lv L, Shao X, Chen H, Ho CT, Sang S. Genistein inhibits advanced glycation end product formation by trapping methylglyoxal. Chem Res Toxicol. 2011; 24: 579-86.
17. Rao AR, Veeresham C, Asres K. In vitro and in vivo inhibitory activities of four Indian medicinal plant extracts and their major components on rat aldose reductase and generation of advanced glycation endproducts. Phytother Res. 2013; 27: 753-60.
18. Wu X, Zhang G, Hu X, Pan J, Liao Y, Ding H. Inhibitory effect of epicatechin gallate on protein glycation. Food Res Int. 2019; 122: 230-40.
19. Liu J, Yang Z, Cheng Y, Wu Q, He Y, Li Q, et al. Eriodictyol and naringenin inhibit the formation of AGEs: An in vitro and molecular interaction study. J Mol Recognit. 2020; 33: e2814.
20. Zielinska D, Laparra-Llopis JM, Zielinski H, Szawara-Nowak D, Gimenez-Bastida JA. Role of Apple Phytochemicals, Phloretin and Phloridzin, in Modulating Processes Related to Intestinal Inflammation. Nutrients. 2019; 11: 1173.
21. Thilavech T, Ngamukote S, Belobrajdic D, Abeywardena M, Adisakwattana S. Cyanidin-3-rutinoside attenuates methylglyoxal-induced protein glycation and DNA damage via carbonyl trapping ability and scavenging reactive oxygen species. BMC Complement Altern Med. 2016; 16: 138.
22. Cao X, Xia Y, Zeng M, Wang W, He Y, Liu J. Caffeic Acid Inhibits the Formation of Advanced Glycation End Products (AGEs) and Mitigates the AGEs-Induced Oxidative Stress and Inflammation Reaction in Human Umbilical Vein Endothelial Cells (HUVECs). Chem Biodivers. 2019; 16: e1900174.
23. Hao M, Li SY, Sun CK, Jingyu X, Lin Y, Liu KX, et al. Amelioration effects of berberine on diabetic microendothelial injury model by the combination of high glucose and advanced glycation end products in vitro. Eur J Pharmacol. 2011; 654: 320-5.
24. Kim JM, Lee YM, Lee GY, Jang DS, Bae KH, Kim JS. Constituents of the roots of Pueraria lobata inhibit formation of advanced glycation end products (AGEs). Arch Pharm Res. 2006; 29: 821-5.
25. J. Magadula J, H. Mbwambo Z, Gatto J, Derbré S, Guilet D, Richomme P. Polyphenolic Compounds with Anti-Ages Activity from Three Clusiaceae Plants. Eur J Med Plants. 2014; 4: 1336-44.
